# Comparative genomics and prediction of conditionally dispensable sequences in legume–infecting *Fusarium oxysporum formae speciales* facilitates identification of candidate effectors

**DOI:** 10.1186/s12864-016-2486-8

**Published:** 2016-03-05

**Authors:** Angela H. Williams, Mamta Sharma, Louise F. Thatcher, Sarwar Azam, James K. Hane, Jana Sperschneider, Brendan N. Kidd, Jonathan P. Anderson, Raju Ghosh, Gagan Garg, Judith Lichtenzveig, H. Corby Kistler, Terrance Shea, Sarah Young, Sally-Anne G. Buck, Lars G. Kamphuis, Rachit Saxena, Suresh Pande, Li-Jun Ma, Rajeev K. Varshney, Karam B. Singh

**Affiliations:** The Institute of Agriculture, The University of Western Australia, 35 Stirling Highway, Crawley, WA 6009 Australia; CSIRO Agriculture, Centre for Environment and Life Sciences, Wembley, WA 6913 Australia; International Crops Research Institute for the Semi-Arid Tropics (ICRISAT), Patancheru, Greater Hyderabad, 502324 Telangana India; Department of Environment and Agriculture, Curtin Institute for Computation, and CCDM Bioinformatics, Centre for Crop and Disease Management, Curtin University, Perth, WA 6102 Australia; Department of Environment and Agriculture, Pulse Pathology and Genetics, Centre for Crop and Disease Management and Curtin Institute for Computation, Curtin University, Perth, WA 6102 Australia; USDA-ARS, Cereal Disease Laboratory, University of Minnesota, St Paul, MN 55108 USA; The Broad Institute, Cambridge, MA 02141 USA; Department of Biochemistry and Molecular Biology, University of Massachusetts, Amherst, MA 01003 USA

**Keywords:** Fusarium, Conditionally dispensable chromosomes (CDC), Effectors, Pathogenicity, Legume, Pulse, Fungal pathogen

## Abstract

**Background:**

Soil-borne fungi of the *Fusarium oxysporum* species complex cause devastating wilt disease on many crops including legumes that supply human dietary protein needs across many parts of the globe. We present and compare draft genome assemblies for three legume-infecting *formae speciales* (ff. spp.): *F. oxysporum* f. sp. *ciceris* (*Foc*-38-1) and f. sp. *pisi* (*Fop*-37622), significant pathogens of chickpea and pea respectively, the world’s second and third most important grain legumes, and lastly f. sp. *medicaginis* (*Fom*-5190a) for which we developed a model legume pathosystem utilising *Medicago truncatula*.

**Results:**

Focusing on the identification of pathogenicity gene content, we leveraged the reference genomes of *Fusarium* pathogens *F. oxysporum* f. sp. *lycopersici* (tomato-infecting) and *F. solani* (pea-infecting) and their well-characterised core and dispensable chromosomes to predict genomic organisation in the newly sequenced legume-infecting isolates. Dispensable chromosomes are not essential for growth and in *Fusarium* species are known to be enriched in host-specificity and pathogenicity-associated genes. Comparative genomics of the publicly available *Fusarium* species revealed differential patterns of sequence conservation across *F. oxysporum formae speciales,* with legume-pathogenic *formae speciales* not exhibiting greater sequence conservation between them relative to non-legume-infecting *formae speciales*, possibly indicating the lack of a common ancestral source for legume pathogenicity. Combining predicted dispensable gene content with *in planta* expression in the model legume-infecting isolate, we identified small conserved regions and candidate effectors, four of which shared greatest similarity to proteins from another legume-infecting ff. spp.

**Conclusions:**

We demonstrate that distinction of core and potential dispensable genomic regions of novel *F. oxysporum* genomes is an effective tool to facilitate effector discovery and the identification of gene content possibly linked to host specificity. While the legume-infecting isolates didn’t share large genomic regions of pathogenicity-related content, smaller regions and candidate effector proteins were highly conserved, suggesting that they may play specific roles in inducing disease on legume hosts.

**Electronic supplementary material:**

The online version of this article (doi:10.1186/s12864-016-2486-8) contains supplementary material, which is available to authorized users.

## Background

*Fusarium* wilt and root rot caused by members of the *Fusarium oxysporum* species complex (FOSC) are major constraints to the production of horticultural, cotton, and legume crops worldwide. *F. oxysporum* is a globally ubiquitous soil-borne fungus [1, 2] and is one of the most important plant-pathogens of the *Fusarium* genus, having been ranked 5th in a list of the top 10 plant pathogens of scientific/economic importance [3]. While some *F. oxysporum* isolates are non-pathogenic saprophytes and may even have symbiotic or bio-control properties [4] this species notably contains more than 150 host-specific plant-pathogenic sub-species [5], known as *formae speciales* (ff. spp. singular forma specialis, abrv. f. sp.). Each of which cause disease on a narrow range of host plant species and which may be further divided into races or pathotypes and additionally vegetative compatibility groups [6].

Many fungi have evolved the ability to attack living plants rather than obtain nutrients saprophytically and the invasion is often facilitated by effector molecules that interact with the host plant’s immune system (reviewed in [7, 8]). In some fungal genera, including *Fusarium,* genes encoding the production of these molecules have evolved on chromosomal regions that are not required for saprophytic growth and are thus known as ‘conditionally dispensable chromosomes’ (CDCs, also known as supernumerary, accessory, lineage-specific, B-chromosomes or mini-chromosomes) in contrast to ‘core’ chromosomes whose gene content is essential and conserved across generations [9, 16]. Dispensable genomic regions encoding genes that play a role in pathogenicity and host-specificity, including effector genes, have been identified in *Fusarium* isolates infecting a range of plant hosts [9–13]. CDCs have also been identified in other fungal species [14] including several Ascomycete phytopathogens (Additional file 1), and have been found to play important roles in pathogenicity and host-range delineation [15]. The first *Fusarium* CDC identified was from *F. solani* (syn. *Nectria haematococca*) and contained a cluster of six pea-pathogenicity (*PEP*) genes involved in detoxifying the plant defence compound pisatin produced by the garden pea, *Pisum sativum* [11–13]. For some *Fusarium* species including the tomato-infecting *F. oxysporum* f. sp *lycopersici* (*Fol*), the genes residing on CDCs define its host range and these chromosomes when transferred to non-pathogenic species can confer pathogenicity on a new host. Dispensable regions of the genome are presumed to be maintained while they convey an evolutionary advantage, either to allow for adaptation of novel genes separately from regions containing core conserved genes, or to allow for the transfer of genetic material e.g. conferring pathogenicity on a new host. The clustering of genes important for pathogenicity on a small CDC chromosome (e.g. as has been demonstrated for CDC 14 of *Fol*) that could be transferred would provide a highly selective advantage for a ‘one step’ horizontal transfer event that could enable an isolate to become pathogenic on a new host [9, 16]. Presumably genes that do not confer pathogenicity on the new host would be more susceptible to shuffling and subsequent loss, as has been observed for *Fol* and *F. solani* CDCs relative to core chromosomes [11].

Genomic mapping and sequencing of *Fusarium* species has revealed chromosome numbers to be highly plastic, ranging from 4–17 [9, 11, 17]. The common ancestor species has been proposed to have only 11 chromosomes, with the increase in chromosome number due in part to the presence of CDCs which are thought to have originated in *F. oxysporum* via horizontal transmission from other *Fusarium* species [9]. Gene content in CDCs is often relatively sparse but enriched in transposable elements. For example, less than 1/8 of *Fol* pathogenicity CDC 14 is predicted to encode protein coding genes and these are predominately proteins of unknown function. In comparison to core chromosomes, CDCs are enriched for pathogenicity-associated proteins, secreted proteins and proteins involved in secondary metabolism [18]. Some *Fol* CDC genes important for pathogenicity encode the SECRETED-IN-XYLEM (SIX) effector proteins [9], and are often associated with distinct repeat types [10, 18]. SIX proteins, first identified in the xylem sap of *Fol-*infected tomato plants, are small, secreted and often cysteine-rich [18, 19]. So far 14 families of SIX proteins have been identified, sharing little similarity with each other or other known fungal proteins (except in *Colletotrichum* sp.- also a member of the class Sordariomycetes). Several have characterised roles in virulence and/or avirulence with their cognate host *R*-genes identified [9, 18–24], although for the most part their biological function within the host remain unknown.

Members of the *Fusarium* genus are major constraints to global grain and forage legume production. *Fusarium* wilts and root rots caused by species such as *F. oxysporum, F. solani, F. udum,* and *F. virguliforme* are a major problem for a number of important legume crops including chickpea, pea, soybean, lentil, lupin, alfalfa, common bean and pigeon pea causing losses upwards of 10 % annually, but in many cases complete loss [25, 26]. These crops provide a high protein food source to a large proportion of the world’s population as well as serving as a source of livestock feed [27]. In addition, they improve the soil through nitrogen fixation and are often used in rotational cropping systems to provide disease breaks.

In this study we generate, inspect and compare the assembled genome sequences and functional annotation of three legume-infecting *formae speciales* of the FOSC, adding to the increasing list of available *F. oxysporum* ff. spp. genome assemblies, with none that infect legumes, the third-largest family of higher plants, previously published. These isolates, *F. oxysporum* f. sp. *medicaginis* (*Fom, Fom-*5190a), *F. oxysporum* f. sp. *ciceris* (*Foc, Foc*-38-1) and *F. oxysporum* f. sp. *pisi* (*Fop, Fop-*37622) are causal agents of *Fusarium* wilt on *Medicago* species (including *Medicago sativa* (alfalfa/lucerne) and the model legume *Medicago truncatula*), chickpea (*Cicer arietinum*) and pea (*Pisum sativum*) respectively. The legume-infecting ff. spp. discussed herein exhibit a similar infection cycle to *Fol*, favoured by warm soil temperatures [28–32]. The hyphae of germinated conidia colonize and penetrate the root epidermis, move inter-cellularly through the root cortex and into the xylem. As shown for the model legume *M. truncatula* in Additional file 2a and for *C. arietinum* in 2b, extensive colonisation of the vascular system leads to water stress, wilting and bleaching of stems and leaves, followed by necrosis and eventually plant death.

Chickpea and pea are the second and third most important legume crops worldwide, with chickpea the most important in India, due to its high protein content (FAO: www.fao.org). *Foc* is a major pathogen of chickpea typically accounting for 10-15 % of yield losses worldwide [33, 34] and can be transmitted via seed [35] but can also survive in the soil for long periods. *Foc* has two known pathotypes, that cause either yellowing or wilt [36], and eight pathogenic races (Races 0, 1, 1B/C, 2, 3, 4, 5 and 6) although it is proposed to be one of the few *F. oxysporum* ff. spp. that is monophyletic [37]. The isolate sequenced in this study *Foc-*38-1, represents the most virulent race of this *forma specialis* (race 1) which shows wide geographic distribution throughout India, the largest producer of chickpeas [38, 39] and is capable of causing complete loss of grain yield [30, 36, 40, 41]. In *Fop* four races 1, 2, 5 and 6 have been described [42] and the isolate sequenced in this study belongs to race 5. *Medicago* spp. are pasture crops typically used for rotation and fodder [43], although alfalfa (*M. sativa*) is also grown for human consumption, and *M. truncatula* is a notable model plant species [44]. The corresponding pathogenic f. sp. (*Fom*) of *M. truncatula* is therefore of relevance as a model for the study of plant-pathogen interactions in legumes while also having bearing on alfalfa/lucerne, the world’s major temperate forage crop [45]. The race of the isolate sequenced in this study *Fom*-5190a is not known, as in this f. sp. races are yet to be defined.

In this work, we focus our analysis on identifying regions of the new legume-infecting *F. oxysporum* genomes that may be relevant to plant pathogenicity in part by predicting potential CD regions and coupling this knowledge with *in planta* expression during *F. oxysporum-* infection of the model legume *M. truncatula.* This process led to the identification of several effector candidates and conserved pathogenicity factors across the legume infecting ff. spp., that we speculate play a role in inducing disease on legume hosts.

## Results and discussion

### Genome features and organisation

The *Fom*-5190a and *Foc*-38-1 genomes were assembled using a combination of paired-end, mate-pair and long-jumping distance Illumina libraries with additional 454 sequencing used for *Fom*-5190a as described in the Methods and Additional file 3. The *Fom*-5190a genome was sequenced at ~170x coverage (trimmed Illumina and 454 data) and assembled into 4034 scaffolds with a total length of ~51.1 Mb and encoding 16,858 proteins. The *Foc*-38-1 genome was sequenced at ~577x coverage (trimmed Illumina data) and assembled into 1482 scaffolds, totalling ~54.8 Mb encoding for 16,124 proteins. The whole-genome sequence of *Fop* NRRL strain 37622 had a final 260× physical coverage generated from two libraries using Illumina sequencing technology. The final assembly encompassed 472 scaffolds with a total length of 55.1 Mb encoding 19,623 genes. Approximately 98 % of highly conserved protein-coding genes were estimated by CEGMA [46] to be represented in all three assemblies, highlighting their comprehensiveness in core regions, and as compared to other *Fusarium* reference assemblies which had similarly high percentages (Table 1). The majority of proteins from all three genomes were functionally annotated based on comparisons to publicly available databases Pfam, InterPro and KEGG (summarised in Additional file 4).

**Table 1:** Genome assembly characteristics and comparisons across *Fusarium* species

	*F. oxysporum* f. sp. *medicaginis*	*F. oxysporum* f. sp. *ciceris*	*F. oxysporum* f. sp. *pisi*	*F. oxysporum* f. sp *.lycopersici* [9]^b^	*F. oxysporum brassica* [57]	*F. solani (syn. Nectria haematococca)* [11]^b^	*F. verticilliodes* (syn. *Gibberella fujikuroi*) [9]	*F. graminearum* (syn. *Gibberell zeae*) [17]
Genome Abbreviation	*Fom*	*Foc*	*Fop*	*Fol*	*Fob*	*Fs*	*Fv*	*Fg*
Isolate	5190a	38-1	NRRL37622	4287	5176	MPVI 77-13-4	7600	PH-1
Chromosome number	Unknown	Unknown	16	15	Unknown	17	11	4
Host	*Medicago spp*.	Chickpea	*Pisum*	Tomato	*Brassica spp.* including *Arabidopsis thaliana*	Pea	Maize and cereals	Wheat and barley
Total assembly length (bp)	51,139,932	54,813,009	55,188,000	57,720,560	54,767,602	47,328,059	41,104,290	36,346,967
Average length (bp)	12,674	36,961	116,923	3,607,535	6969	2,629,337	3,425,358	7,269,393
Maximum length (bp)	4,225,797	2,918,844	5,895,957	6,854,980	415,898	6,369,736	6,219,215	11,694,295
Minimum length (bp)	200	1000	2000	1,646,460	200	215,166	2,040,847	7,711,129
L50 (bp)	1,632,076	182,371	2,840,000	4,589,937	60,340	3,621,839	4,236,349	8,911,601
N50	10	58	54	6	222	6	5	2
Total sequences	4034	1482	472	15	7858	17	11	4
Total unknown bases (N)	1,929,171	2,262,803	12,221	1,238,430	33	1915	87,573	212,843
GC%^c^	48.2	48.1	47.6	48.5	47.8	51.5	48.7	48.4
Percentage of *de novo* predicted repetitive sequences^c^	1.5	5.4	8.2	21.9	9.4	3.1	1.3	1.5
Percentage of predicted repetitive sequences based on comparison to known fungal repeats in Repbase	1.0	2.4	2.8	4.4	2.4	1.4	0.8	0.9
CEGMA Analysis^a^								
CEGMA partial %	98	98	98	97	98	98	96	97
CEGMA partial	243	244	242	241	243	243	239	241
CEGMA complete	239	240	237	237	238	238	235	234

^a^Number of complete Conserved Eukaryotic Genes found in the assembly of the core set of 248 defined by Parra et al. [46]

^b^Reconstructed chromosome mapped regions only

^c^GC content and d*e novo* repeat percentage based on RepeatMasker analysis of scaffolds versus *de novo* repeats

The most in-depth studied genome of an *F. oxysporum* ff. spp. to date belongs to *Fol*, which was assembled into near-complete chromosome sequences via an optical map [9]. We therefore used *Fol* as our primary point of reference for subsequent comparative genomics. In some analyses we have also made additional comparisons to the similarly high-quality chromosome assembly of *F. solani* [11] which is a more distantly related species but shares some legume hosts with the novel isolates presented here. The main protein features of *Fom*-5190a, *Fop*-37622 and *Foc*-38-1 are compared in Table 2 with those of other *Fusarium* species which shows that *Fom*-5190a and *Foc*-38-1 had similar gene numbers despite their differing assembly sizes (*Fom*-5190a 51.1 Mb versus *Foc*-38-1 54.8 Mb), which appear to be influenced mostly by repetitive DNA content (Table 1). The number of small secreted proteins (SSPs), indicative of putative roles as effectors, across the legume-infecting ff. spp. was comparable to those predicted in the two other *F. oxysporum* ff. spp. genomes analysed (*Fol* and *Fob*-5176), using the criteria of protein length ≤ 300 amino acid, predicted to be secreted and containing ≤ one transmembrane domain in the N-terminal region.

**Table 2:** Protein set comparisons across *Fusarium* species

	*Foc*	*Fom*	*Fop*	*Fob*	*Fol*	*Fsol*	*Fg*	*Fv*
Total number of proteins	16,124	16,858	19,623	17,817	17,701	15,707	13,321	20,553
SSPs^a^	537	580	620	588	597	426	483	621
Number of species specific proteins (including paralogs)	1090	863	832	1606	1825	2083	1632	1852
Number of unique species specific proteins^b^	1058	808	785	1540	1645	1919	1622	1687

^a^Small secreted proteins

^b^No orthologs or paralogs in any of the other 43 spp. tested including all publicly available *Fusarium* spp.

Isolate details are as detailed in Additional file 5

*Foc* *F. oxysporum* f. sp. *ciceris*, *Fom* *F. oxysporum* f. sp. *medicaginis,* *Fop F. oxysporum* f. sp. *pisi,* *Fob F. oxysporum 5176,* *Fol F. oxysporum* f. sp. *lycopersici,* *Fsol F. solani,* *Fg F. graminearum,* *Fv F. verticilliodes*

To expand our analysis and aid identification of proteins common to the legume-infecting ff. spp., we next conducted protein orthology comparisons across 44 diverse fungal species (Additional file 5) both closely and distantly related but sharing similar hosts (Additional file 6). This analysis identified 1090 paralog groups unique to *Foc*-38-1, 823 in *Fop*-37622 and 863 unique to *Fom*-5190a (containing 1–5 paralogs per gene) (Table 2). Those genes that do not have orthologs in another ff. spp. are presumed likely to encode proteins that play a role in host specificity, and were used in subsequent analyses predicting effectors. However, host specificity may also be governed by small differences in orthologous proteins that may affect their interaction with host protein. There were 10,602 ortholog groups shared by the legume-infecting ff. spp. (*Fom, Fo*c and *Fop*) of which 8118 were also shared with the legume pathogen *F. solani.* A similar number of *F. solani* sequences (over 9000) was observed to be conserved within three distinct species of the genus *Fusarium* (*Fol*, *F. graminearum* and *F. verticilliodes* [9]) suggesting that this corresponds roughly to the number of core genes conserved amongst *Fusarium* species.

We next examined G:C content in the new genomes as G:C variation in other fungal phytopathogens has highlighted key pathogenicity regions. For example, *Leptosphaeria maculans* (blackleg disease, stem canker of *Brassicas*) has AT-rich isochores throughout its core genome [47], while *Z. tritici* (septoria leaf blotch of wheat) possesses AT-rich CDCs [48]. However we found that G:C content across the legume-infecting ff. spp. scaffolds, as well across chromosomes of the *Fol* reference, were relatively constant at ~46–48 % with, in general, no large regions of atypical G:C % (isochores) observed, even within *Fol* CDCs. There were however small local variations on CDCs around transposons and other repeated sequences, resulting in a marginally lower chromosome average G:C% for core versus dispensable scaffolds of *Fol* and *F.* s*olani* which was also observed for predicted dispensable versus core scaffolds from the legume-infecting ff. spp. (Additional file 7).

Next a comparison of repetitive DNA content across *Fusarium* spp. was conducted as it is an important feature in many fungal genomes, driving evolution through repeat induced point mutation, gene duplication or altering gene expression through insertion into or near other genes [49–51]. Prediction of repetitive DNA in assemblies that primarily use short-read next-generation sequencing data, such as the recently assembled *Fusarium* spp. genomes, is generally underestimated compared to their Sanger-based counterparts (e.g. *Fol*-4287) due to the presence of repeats in unassembled sequences. Consequently the *de novo* predicted repeat content of the novel *Foc*-38-1, *Fop*-37622 and *Fom*-5190a genomes was considerably lower than that of *Fol* (Table 1) although, the repeat content of other *F. oxysporum* ff. spp. sequenced and assembled using similar methods and analysed via this method were found to be within similar ranges (3.9–9.4 %, Additional file 8a).

In *Fol* CDCs, DNA transposons were over-represented and Ma et al. (2010) speculate that their expansion in *Fol* may correlate with the formation of the *Fol* lineage-specific regions as well as segmental duplications of some regions of the genome [9]. Additionally, the predicted effector genes of *Fol,* and the *F. solani* and *F. oxysporum* f. sp. *betae* pea pathogenicity (*PEP*) clusters have been observed to reside within genomic subregions enriched with DNA transposons [9, 18, 52, 53]. To aid localisation of pathogenicity-associated gene content, we therefore scanned the legume-infecting *F. oxysporum* ff. spp. genomes for transposable elements (TEs). The *Foc*-38-1 and *Fop*-37622 assemblies contained a larger number of DNA transposons and retrotransposons than *Fom*-5190a (Additional file 8b), with the majority of predicted TEs in all three belonging to the *Tc1-mariner* superfamily of DNA transposons which includes the *impala* family [54]. Remnants of *impala* and *Fot5* transposons have been observed to occur in the vicinity of several *Fol SIX* effector genes [18, 22] and have been used to predict new effector-like genes in *Fol* and *F. oxysporum* f. sp. *melonis* [10, 18].

### Known conditionally dispensable chromosomes in *Fusarium* spp. exhibit varying sequence conservation across legume-infecting isolates and other *Fusarium* spp.

**Fig. 1:**
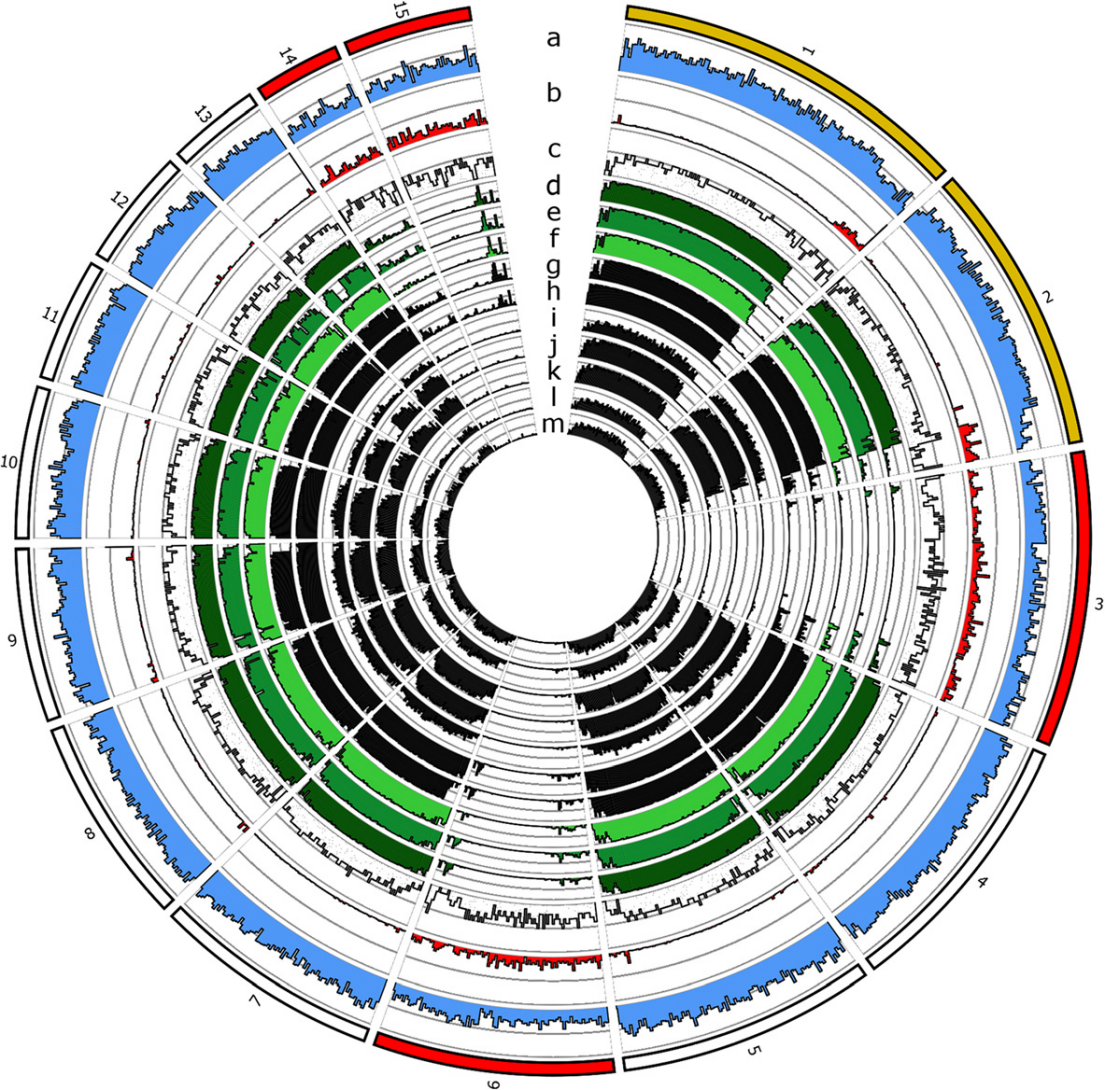
*F. oxysporum* f. sp. *lycopersici* chromosomes highlighting sequence conservation and other features of CDCs in comparison to core chromosomes. The circos plot illustrates the gene-sparse, repeat-rich nature of *Fol* CDCs and their lower sequence conservation across related species in comparison to core chromosomes. Outer ring-*Fol* chromosomes highlighting CDCs (red) and chromosomes that are partially dispensable (yellow). Inner rings: (*a*) gene density in 100 kb windows, (*b*) repeat density in 100 kb windows, (*c*) GC content in 50 k bp windows, range 45–55 %, (*d*) Region of *Fol* chromosomes overlapped by *Fom*-5190a sequences, (*e*) *Foc*-38-1, (*f*) *Fop-*37622, (*g*) *F. oxysporum* f. sp. *brassica Fo5176*, (*h*) *F. oxysporum* f. sp. *melonis,* (*i*) *F. solani,* (*j*) *F. fujikuori,* (*k*) *F. verticilliodes,* (*l*) *F. virguliforme*, and (*m*) *F. graminearum*

CDCs of *Fol* are thought in part to define host range, are enriched for effectors and can be transferred to non-pathogenic species to confer pathogenicity [1]. We therefore aimed to isolate lineage specific gene content in the legume-infecting isolates by identifying their potential CDC sequences. To do this we aligned their scaffold sequences to the non-repetitive regions of chromosomes of *Fol* and *F. solani* in which CDCs are well defined [9, 11], as well as those of other publicly available *Fusarium* spp. to compare the levels of conservation. The chromosome sequences of *Fol* [9] and *F. solani* [11] were masked based on the presence of *de novo*-predicted repetitive DNA sequences and then translated and aligned to the repeat-masked genomes of other *Fusarium* spp. using MUMmer [55]. A distinct pattern of variation in levels of sequence conservation between core and dispensable chromosomes was observed across *F. oxysporum* ff. spp. and other *Fusarium* spp. (Fig. 1). Similar patterns were also observed for *F. solani* chromosomes (Additional file 9). The percentage length of each *Fol* and *F. solani* chromosome (excluding masked repetitive sequences) that was covered by one or more matches is summarised in Additional file 10a and b.

Presence-absence variation relative to chromosomes of *Fol* indicated a distinctive pattern of widespread absence of sequences homeologous to *Fol* CDCs 3, 6, 14 and 15 across *Fusarium* spp. as previously described [9]. These *Fol* CDCs are distinct from core chromosomes in having markedly higher repetitive DNA content (as determined by the total length of sequences masked as *de novo*-predicted repeats). For the legume-infecting isolates an average of ~93 % sequence conservation to the masked *Fol* core scaffolds was observed but only ~25 % for the CDCs (Additional file 10a). Partial conservation of the non-repetitive sequences of *Fol* pathogenicity CDC 14 (42–51 %) was observed in *Foc*-38-1 and *Fom*-5190a, as well as the *Arabidopsis*- and melon-infecting isolates *Fo5176* and *Fom-*26406 respectively, however similar levels of conservation were not observed in *Fop*-37622 or across other publicly-available *F. oxysporum* ff. spp. genomes (BROAD MIT, Additional file 10a). This observation is interesting as most CDCs were initially thought to lack homology or synteny to related species [9, 11, 56], although consistent with the finding that *Fo5176* shared an average of 34.5 % of the total sequence of the *Fol* CDCs (described in supplementary data presented in [9]) and that *SIX* genes originally thought to be unique to *Fol* have been identified in several other ff. spp. [57–62]. The fact that *Foc*-38-1 and *Fom*-5190a show greater sequence conservation of *Fol* CDC 14 (51 % and 42 % respectively) than *Fop-*37622 which shares only 20 % non-repetitive sequence indicates that legume infecting ff. spp. may not derive their pathogenicity from common sequences conserved with *Fol.* The length of conserved sequence with *Fol* pathogenicity CDC 14 was also low in several other *F. oxysporum* ff. spp. including unsurprisingly the non-pathogenic biocontrol species (Fo47, 8 %) as well as pathogens of other plant species (less than 20 % for pathogens of tomato and banana, ff. spp. *radicis lycopersici* and *cubense*). Although conservation as high as 88 % was observed in another tomato-infecting isolate and 45–50 % in *F. oxysporum* f. sp. *conglutinans* and *F. oxysporum* f. sp. *raphani*. Similar patterns of variation in conservation across ff. spp. were also observed for other *Fol* CDCs in isolates included in this study (Additional file 10).

We postulated that pathogenicity on legumes may be due to conserved CD sequences within the legume-infecting ff. spp. and possibly shared with the legume pathogen *F. solani.* A presence-absence variation analysis relative to *F. solani* chromosomes found distinct absences across *Fusarium* spp. for known CDCs 14, 15 and 17 - all of which have distinctively high repetitive content relative to core chromosomes (Additional files 9 and 10b). Genes important for *F. solani* pathogenicity on legumes are known to be encoded on CDC 14 which is proposed to have been acquired via horizontal transfer [11]. This includes the *PEP* cluster identified in *F. solani* mating population IV [63]. Several genes from this cluster are thought to have been transferred to *F. oxysporum* f. sp. *pisi* [64], with orthologs of four genes demonstrated to contribute to virulence on pea identified in this f. sp. (*PDA1, PEP1, PEP2* and *PEP5*). Yet the non-repetitive sequence of *F. solani* CDC 14 shared only 17 % sequence conservation with *Fop*-37622, 16 % with *Fom*-5190a*,* and 21 % with *Foc*-38-1, with similar values also observed in the non-legume pathogens *Fo5176* (brassicas) and *Fom-*26406 (melon)*.* Therefore *F. solani* appears not to be the common origin for legume-infecting ff. spp. pathogenicity content, although we did identify orthologs of some *PEP* genes with known roles in virulence as discussed in later sections.

### Phylogenetic and orthology analysis indicates independent origins of legume host- specificity

One possible explanation for the evolution of legume-host specificity in *F. oxysporum* isolates is a common ancestor shared by only legume-infecting ff. spp., however this was not supported by a phylogenetic analysis of 100 randomly selected orthologous genes across *Fusarium* spp. (Additional file 11). Thus legume-pathogenicity appears to have arisen more than once within this species. Another possibility is that the legume-infecting ff. spp. may share a set of similar proteins governing legume-host pathogenicity, arrived at via either lateral gene transfer or convergent evolution. However, based on the orthology analysis, only one protein was common to just the four legume-infecting *Fusarium* species and the encoding gene was not detected as expressed under the *in planta* conditions examined in the following sections (Additional file 12).

### Comparative analysis of predicted core and dispensable sequences in legume-infecting isolates and their gene content with other *F. oxysporum* ff. spp.

Drawing upon sequence comparisons to core chromosomes and experimentally demonstrated CDCs of *Fol* and *F. solani* [9, 11], scaffolds from *Fom*-5190a, *Fop*-37622 and *Foc*-38-1 were predicted as either “core” or putatively “dispensable” (Additional file 13a, b and c). Scaffolds with unique matches across more than 30 % of their length to core chromosomes in *Fol* or *F. solani* were designated “core” scaffolds, whilst those with no match, or that matched to a CDC in either genome, were designated as potentially “dispensable”. In general we observed that the newly predicted conditionally dispensable sequences of the legume-infecting ff. spp. shared known characteristics of *Fusarium* CDCs. They had increased repetitive content, reduced gene density, smaller average size of predicted proteins relative to those encoded on core scaffolds and a slightly lower average G:C% (Table 3). The predicted dispensable scaffolds were also more numerous and shorter in average length than those scaffolds predicted to form part of core chromosomes (Additional file 14), presumably due the influence of repetitive sequence on the assembly of those genomic regions.

**Table 3:** Properties of scaffolds predicted to form part of either core or dispensable chromosomes in legume-infecting *formae speciales*

	*Fom*-5190a core	*Fom*-5190a dispensable	*Foc*-38-1 core	*Foc*-38-1 dispensable	*Fop-*37622 core	*Fop-*37622 dispensable
Number of scaffold sequences	446	3529	413	1069	116	356
Total length (bp)	42,167,838	8,766,042	41,518,444	13,294,565	43,587,736	11,600,480
Average length (bp)	94,547	2484	100,529	12,436	375,756	64,988
Length gene coding (bp)	23,149,588	2,802,952	21,935,481	4,117,674	23,633,062	4,042,338
Length repetitive (bp)	277,400	234,489	707,976	580,264	1,354,046	3,172,535
% Gene coding	54.90 %	31.98 %	52.83 %	30.97 %	54.2 %	34.8 %
% Repetitive	0.66 %	2.67 %	1.71 %	4.36 %	3.1 %	27.3 %
Number of predicted genes	14,427	2424	12,985	3139	16,405	3218
Percentage of genes with Pfam annotaion	71 %	48 %	72 %	47 %	66 %	51 %
Gene density (per 10 Kb)	3.4	2.8	3.1	2.4	3.7	2.7
Average protein length (aa)	479	330	486	361	443	374

In order to facilitate the search for genomic regions with roles in plant pathogenicity we compared the sequences of predicted dispensable scaffolds from the legume-infecting ff. spp. with CDCs of *Fol* and *F. solani* or other *F. oxysporum* ff. spp. to identify those with high conservation levels. A comparison between the predicted CD sequences of *Fom*-5190a and *Foc*-38-1 revealed that although the total size difference between the predicted dispensable regions was 4.5 Mb (Table 4), the length of conserved non-repetitive sequence between these isolates was very similar (~3.1 Mb). We speculate that increased repetitive content in the predicted *Foc-*38-1 dispensome is reflective of and potentially accounts for its overall increased assembly length. Interestingly, in contrast to the phylogenetic studies based on genes encoded on “core” scaffolds (Additional file 11), after masking repetitive and low complexity sequences *Foc*-38-1, *Fop*-37622 and *Fom*-5190a predicted dispensable scaffolds shared highest sequence conservation with the *Brassica*-infecting isolate *Fo5176* (34.7, 38.3 and 44.8 % respectively), closely followed by the melon-infecting *F. oxysporum* f. sp. *melonis* (NRRL 26406) and the other legume-infecting isolates. The masked predicted dispensable scaffolds of *Foc*-38-1 and *Fom*-5190a share a greater length of conserved sequence with the pea-infecting isolate *Fop*-37622 than with each other (Table 4). No long runs of consecutive conserved genes between the ff. spp. were observed in predicted dispensable sequences although this may be due to the fragmented assembly of these repeat-rich genomic regions. These data suggest that the legume-infecting ff. spp. may not have acquired and retained whole chromosome sized segments of CD content specific to pathogenicity on legume hosts. They may however, share smaller conserved segments.

**Table 4:** Summary of sequence conservation between *Fom*-5190a and *Foc*-38-1 predicted dispensable scaffolds and other *Fusarium* species

		*Foc*-38-1	(Masked)^a^			*Fom*-5190a	(Masked)^a^			*Fop*-37622	(Masked)^a^	
Total length of predicted dispensable scaffolds (bp)		13,294,565	(12,714,301)			8,766,042	(8,531,553)			11,600,480	(8,427,945)	
	Foc length conserved	Foc sequence conserved	Foc length conserved masked^a^	Foc sequence conserved masked	*Fom* length conserved	*Fom* sequence conserved	*Fom* length conserved masked	*Fom* sequence conserved masked	*Fop* length conserved	*Fop* sequence conserved	*Fop* length conserved masked	*Fop* sequence conserved masked
	(bp)	(%)	(bp)	(%)	(bp)	(%)	(bp)	(%)	(bp)	(%)	(bp)	(%)
*Foc-*38-1	-	-	-	-	3,504,836	40	3,051,034	34.8	5,820,869	50.2	3,747,704	32.3
*Fom-*5190a	3,618,892	27.2	3,134,089	23.6	-	-	-	-	5,200,782	44.8	3,852,673	33.2
*Fop-*37622	5,645,201	42.5	4,105,088	30.9	4,413,435	50.3	3,484,062	39.7	-	-	-	-
*F. solani*	1,417,711	10.7	1,099,653	8.3	1,215,395	13.9	1,037,655	11.8	1,458,181	12.6	954,748	8.2
*F. oxysporum* f. sp*. lycopersici*	4,580,988	34.5	2,041,608	15.4	3,174,649	36.2	1,543,921	17.6	5,107,489	44.0	1,636,670	14.1
*F. oxysporum* f. sp*. melonis*	5,635,252	42.4	4,242,674	31.9	4,684,280	53.4	3,907,121	44.6	6,686,394	57.6	3,999,585	34.5
*Fo*5176	6,429,341	48.4	4,618,139	34.7	5,013,128	57.2	3,930,676	44.8	7,604,002	65.5	4,446,346	38.3

^a^Masked sequences exclude repetitive regions

As CDCs from *Fusarium spp.* are known to be enriched for pathogenicity-associated genes (e.g. including those encoding cell-wall degrading enzymes, *SIX* effectors and other effector-like proteins, transcription factors, and proteins involved in signal transduction and lipid metabolism), but lack ‘housekeeping’ genes [9], we next compared the gene content and assigned functions of genes encoded on the predicted dispensable scaffolds with that of predicted core scaffolds. A larger proportion of the proteins encoded on scaffolds assigned as dispensable had no known function based on Pfam annotation (Table 3). Over half of the manually-curated non-TE ORFs from *Fol* CDC 14 also have no known function [18], highlighting one of the main obstacles in assigning biological roles to potential pathogenicity genes due to the lack of conserved domains identified in most fungal effectors, which thus require experimental evaluation. For genes assigned functional annotation based on comparisons to the Pfam database [65], most functional groups enriched on predicted dispensable scaffolds were similar to those observed on *Fol* CDCs [18] (details in Additional files 15, 16 and 17). These included: Major Facilitator Superfamily (MFS) transporters, transcriptional regulators, sugar transporters, methyl transferases, chitin-binding domains (LysM), p450s, HET domains (with possible roles in vegetative compatibility that may influence potential sequence transfer), NACHT domains (may be associated with proteins involved in heterokaryon incompatibility-HET-E in *P. anserina,* or apoptosis [66]), and Carbohydrate-Active Enzymes (CAZymes: (GH3 and GH43) [67, 68]) as well as several peptidases. Additionally in *Foc*-38-1 and *Fop*-37622 an enrichment of TE related ORFs were observed relative to predicted core scaffolds, including those with domains related to reverse transcription, transposition, DNA binding and dimerisation (Additional files 16 and 17), which is consistent with their elevated repeat content relative to *Fom*-5190a.

### Expression of predicted-CD sequence-encoded genes in *Fom*-5190a with conservation across legume-infecting *F. oxysporum* ff. spp.

In order to further narrow in on predicted CD scaffold gene content with potential roles in pathogenicity that may be conserved within the legume-infecting ff. spp., we used our model *Fom*-5190a legume pathosystem to identify genes expressed during an early stage of infection. From RNA sequencing of three pooled biological replicates of infected *M. truncatula* roots at 2 days post inoculation (dpi) we found that for each replicate 0.1, 0.08 and 0.07 % of the reads mapped to the *Fom*-5190a assembly respectively, giving combined support for the expression of 6448 genes (out of 16,858 predicted). Due to the early time-point and the low fungal biomass at this stage of infection, only 201 genes had 100 % coverage of the predicted gene model with RNA-seq reads, with 1181 genes having 50 % or more coverage, and the remaining overlapped by one or more reads. Of the 6448 genes with expression data, 367 genes were encoded on predicted *Fom*-5190a dispensable chromosomes.

Comparison of predicted *Fom*-5190a dispensable scaffolds that shared greater than 40 % sequence identity with the pea pathogen *F. solani* identified 87 scaffolds encoding 102 genes, 16 of which were expressed by *Fom*-5190a at 2 dpi. These included genes with potential roles in pathogenicity such as cytochrome p450s, glycoside hydrolases (GH28), peptidases, a sodium/hydrogen-exchanger family protein, and fungal transcription factors.

The same comparison between *Fom*-5190a dispensable scaffolds and *Foc*-38-1 identified 176 genes expressed at 2 dpi. This included several small clusters of 3–4 genes expressed *in planta*, on scaffold 29 (19 genes), scaffold 24 (29 genes) and a cluster of ‘restless-like’ transposons on scaffold 124. TEs have been previously observed to be active in several *F. oxysporum* genomes [9, 69], and many in *Fom*-5190a were also found to be transcribed during infection. This indicates they are still active and may be involved in the rearrangement of the genome. The genes encoded on scaffold 29 and the smaller gene clusters predominantly encoded proteins of unknown function but also included fungal transcription factors, proteases, peptidases, and MFS and ABC transporters. *Fom*-5190a scaffold 24 is ~380 kb in length, encodes 142 genes and many of those detected as expressed *in planta* (29) have possible associations with pathogenicity, including pectate lyases, MFS transporters, peptidases, cytochrome P-450 s, and components of the *F. solani PEP* cluster (proteins with similarity to the pisatin demethylase *PDA1,* two other *PDA*s and *PEP5*) [52]. However, this scaffold shares only 23 % sequence conservation with *F. solani,* indicating that there has not been a large scale transfer of the CDC, or that if there was, this region of the *Fom*-5190a genome has since undergone significant reshuffling. *Fom*-5190a scaffold 24 does however share 71.8 % sequence similarity with *Foc-*38-1 and 83.5 % with *Fop-*37622, suggesting that these sequences may share a similar source. This scaffold also shares 87 % sequence conservation with *Fo5176* and *F. oxysporum* f. sp. *melonis,* but only 12.7 % with *Fol.* The source and route of transfer amongst *F. oxysporum* ff. spp. of the CDC that this scaffold is thought to represent, may become apparent with further comparative studies enabled by the growing number of available *Fusarium* genomes.

Finally, a comparison between *Fom*-5190a predicted dispensable scaffolds and *Fop-*37622 identified two scaffolds with high sequence similarity. Firstly, *Fom*-5190a scaffold 31 shared more than 183 kb (88.4 %) with *Fop*-37622. This scaffold encodes 82 genes including several MFS transporters, a cytochrome p450 and several fungal transcription factors. Twelve of these genes were expressed at 2 dpi, with three predicted to be secreted, and four consecutively encoded (*FOXM-5190a_14251-14254*) including those with similarity to a FAD dependent oxidoreductase, a protein-arginine deiminase and an MFS transporter as well as a nearby encoded isochorismatate hydrolase. This scaffold also shares over 86 % homology with *F. oxysporum* f. sp. *melonis* and *Fo5176* but not *Foc-38-1, F. solani* or *Fol*, suggesting it hasn’t been derived from a common legume-infecting isolate. Another *Fom-*5190a scaffold, scaffold 113, shares high sequence conservation with only *Fop*-37622 (80 % versus ~30 % in the other ff. spp. compared) and encodes 7 genes (*FOXM_5190a_15729-15735*) all of which were expressed *in planta* at 2 dpi including a fungal transcription factor, an ABC transporter, a monoxygenase (FAD_binding_3) and a glutathione S-transferase. Four of these genes (*FOXM_5190a_15729-15732*) are also co-linear with *Fop*-37622 genes (*FOVG_17777-17780*). The properties of these genes: location on predicted CD sequences, expression early in infection and conserved synteny, collectively suggests important roles in the infection process and thus these genes will be prioritised for follow up in future functional studies.

### Proteins with potential roles in legume phytoalexin detoxification

As orthologs or possible components of the *F. solani PEP* cluster were identified several times in the above analysis, we investigated this cluster in further detail. Legumes are known to produce low molecular weight antimicrobial compounds, known as phytoalexins including maackiain in chickpea, pisatin in pea, and medicarpin in *Medicago* sp. [70, 71]. These pterocarpan molecules are structurally similar and are toxic to several genera of fungi and legume pathogens with the ability to detoxify or export these compounds are more virulent [72–75]. In the pea pathogens *Fop* and *F. solani* the phytoalexin pisatin is demethylated by a cytochrome P450 known as pisatin demethylase (PDA) shown to be important for virulence on this host [64, 71, 76] and demonstrated to be able to detoxify pisatin in isolation [77, 78]. In *F. solani* the *PEP* cluster of genes containing *PDA1* is found in the reference mating population on CDC 14 and exhibits altered codon usage compared to core genes [52]. The cluster contains six genes in *F. solani*, four of which have demonstrated roles in virulence (*PEP1, 2*, *5* and *PDA1*) [52], all of which are induced in response to pisatin and during infection of pea [79] and function independently as virulence factors [52, 79]. Apart from *PDA1* only two have proposed biological functions *PEP5* is a potential MFS and *PEP2* possibly has a role in RNA binding [52]. Our analysis showed that none of the legume infecting *F. oxysporum* ff. spp. contained the *PEP* cluster in its entirety. *Fop*-37622 has two copies of *PEP2* (*FOVG_17451T0, FOVG_16839T0*), whilst *Foc*-38-1 has one (*FOC38_09209*) and *Fom*-5190a has none. Genes similar to *PEP5* and containing MFS domains [Pfam:PF07690.11] were found in *Fop* (*FOVG_16838T0*), *Foc*-38-1 (*FOC38_09210*), and *Fom*-5190a (*FOXM*_*5190a_13563, FOXM*_*5190a_15270*) where *FOXM_5190a_13563* was next to a *F. solani PDA1* ortholog. We identified two orthologs of *PDA1* in *Foc*-38-1 and four in *Fom*-5190a, three of which were detected as expressed *in planta* (Additional file 18). Several of these orthologs had greater homology to *F. solani PDAs*, while the others were closer to *PDA* genes from *Fop.* Previous analyses of *F. oxysporum* f. sp. *pisi* isolates showed that while homologs of genes from the *F. solani PEP* cluster are often present amongst isolates of the different *Fop* races, their location can differ across races and they are rarely identified as a cluster [64], supporting the idea of multiple origins for *Fop* races unrelated to *PDA* gene content [64]. These studies also show that orthologs of *PDA1* are present within a group of related ff. spp. that are pathogenic on dicots (f. sp. *lini, pisi, dianthi*) although the encoded proteins are not always functional due to small but important amino acid changes [64]. A functional homolog, demonstrated to be more closely related to *Fop PDA1* than *F. solani PDA1,* was identified in f. sp. *phaesoli* (cause of wilt on common beans, *Phaseolus vulgaris*) which was also virulent on pea and that the authors suggest may have arisen via HGT [64]. Another possible explanation proposed in a recent study [80] is that *PDA1* is vertically inherited within the FOSC, rather than via HGT from *F. solani* as previously proposed [53, 81].

The *F. solani PEP* cluster also contains *Nht1* transposons [82], however none were found in the genome assemblies of *Foc*-38-1*, Fom*-5190a or *Fop-*37622*,* another possible indicator of the separate evolution of these genes in these ff. spp. from a common ancestor rather than transfer of a whole region. It is possible that some of the genes characterised as *PDAs* are detoxifying pterocarpans other than pisatin, such as medicarpin or sativan which are produced by both alfalfa and *M. truncatula.* However further investigation including biochemical examination of the breakdown products of these fungal enzymes will be required to determine this.

### Prediction of effector genes in *Fom*-5190a– pathogen of the model legume *Medicago truncatula*

Like many other plant pathogens, *Fusarium* spp. are known to produce small secreted proteins and secondary metabolites to manipulate and evade their host plant’s defences [83, 84]. In addition to identification of proteins with known roles in plant pathogenicity in other fungal species (Additional file 18) a combination of multiple sources of evidence was used to predict putative legume host-specific effectors. This incorporated predictions of secretion and protein size, orthology across *Fusarium* spp. and orthology-based lineage-specificity, functional annotations, predictions of dispensable sequences, proximity to pathogenicity gene-associated repetitive DNA, and RNA-seq data derived from our model legume pathosystem.

We identified 580 SSPs in *Fom*-5190a, and 537 and 620 respectively in *Foc*-38-1 and *Fop*-37622 (Table 2, Additional files 19, 20 and 21). This number is comparable to that predicted in other *Fusarium oxysporum* ff. spp. and *Fusarium* spp. (Table 2). In *Fom*-5190a, 75 SSPs were predicted to occur on potentially dispensable scaffolds, with 94 and 98 respectively found in *Foc*-38-1 and *Fop*-37622. RNA-seq data from infected *M. truncatula* roots showed that 19 of these SSPs were expressed at 2 dpi. This included four homologs of the *Fol SECRETED IN XYLEM (SIX)* genes, with proposed roles in virulence/avirulence on tomato (*SIX1, SIX8, SIX9, SIX13*). Of these 19 proteins, only five had characterised Pfam domains including a GH16, CFEM and LysM, a peroxidase and a DUF3129 domain (Table 5, Additional file 19).

**Table 5:** Properties of *Fom-*5190a effector candidates

Gene	*FOXM-5190a_15788*	*FOXM-5190a_16235*	*FOXM-5190a_16257*	*FOXM-5190a_16301*	*FOXM-5190a_16306*	*FOXM-5190a_16326*	*FOXM_5190a-SIX1*	*FOXM_5190a-SIX8*	*FOXM_5190a-SIX9*	*FOXM5190a-SIX13*
Protein length (aa)	199	264	91	144	131	111	279	141	122	292
Mature protein length (aa)	186	247	72	128	112	95	263	126	105	276
Cysteine count in mature protein	7	8	8	6	10	9	8	2	6	6
% Cysteine (of aa length)	3.5	3	8.8	4.2	8.9	8.1	2.8	1.4	5.7	2.1
Predicted to be secreted by SignalP	Y	Y	Y	Y	Y	Y	Y	Y	Y	Y
WoLFPsort predicted location	extr	extr	extr	extr	extr	extr	extr	extr	extr	extr, mito
Phobius predicted signal peptide	Y	Y	Y	Y	Y	Y	Y	Y	Y	Y
TMHMM predicted transmembrane count	0	0	0	0	0	0	0	0	0	0
Pfam domains	-	-	-	LysM	-	-	-	-	-	-
Number of aligned RNAseq reads overlapping gene model from combined 2d *in planta* libraries	24	1039	89	378	10	369	2546	828	246	440
Average depth of RNAseq coverage per base of gene	4	123	27	88	3	76	354	141	33	61
Gene length (bp)	655	844	324	432	458	488	720	587	728	720
Proportion of gene model supported by RNAseq	0.96	1	1	1	0.69	1	0.99	1	1	1
Scaffold	122	322	364	461	485	531	3157, 696, 1167	420	270	306, 3812
Scaffold length (bp)	28,293	2356	1489	971	914	815	219, 647, 416	1129	4106	3,471, 204
Predicted scaffold type	Dispensable	Dispensable	Dispensable	Dispensable	Dispensable	Dispensable	Dispensable	Dispensable	Dispensable	Dispensable
Orthology	Has orthologs in other ff.spp.	Has orthologs in other ff.spp.	Single ortholog unique to *Fom*5190a	Has orthologs in other ff.spp.	Has orthologs in other ff.spp.	Has orthologs in other ff.spp.	Has orthologs in other ff.spp.	Has orthologs in other ff.spp.	Has orthologs in other ff.spp.	Has orthologs in other ff.spp.

Further manual inspection of the annotation and level of RNA-seq expression of the 19 SSPs encoded on *Fom-*5190a dispensable scaffolds identified a subset of 10 genes that were prioritised for further initial investigation as effector candidates (which included the *SIX* gene homologs) (Table 5). Protein orthology analysis supported only one of these proteins (FOXM_5190a_16257) as lineage-specific to *Fom*-5190a with the others sharing best BLASTP matches of 42–99 % similarity to other *F. oxysporum* or *F. fujikuroi* proteins (Additional file 22). Of the SIX homologs, FOXM_5190a_SIX1 was most similar to that identified in *Fop*-37622 (87 %) and the FOXM-5190a_SIX8 best BLASTP match was FOC-38_SIX8, differing by only one amino acid (99.3 % identity) suggesting that these genes, in particular SIX8, may have a common origin. *Fom*-5190a SIX9 and SIX13 homologs shared less conservation to legume pathogenic ff. spp., with best matches to *Fol* (42 %) and *F. oxysporum* f. sp. *melonis* (74 %) respectively. Two other effector candidates FOXM_5190a_16301 and FOXM_5190a_16306, which contain no characterised domains, were also most similar to proteins from legume-infecting ff. spp., *Fop*-37622 (95 %) or *Foc*-38-1 (99 %) respectively. While these ten effector candidates constitute a shortlist for further investigation, the overall *Fom*-5190a pathogenicity gene set may be much larger with 183 of the 580 predicted SSPs detected as expressed *in planta* at 2 dpi.

Because our analysis highlighted the potential importance of SIX gene homologs during *Fom-*5190a infection we searched for these proteins in the other *F. oxysporum* ff. spp. and sp. (summarised in Table 6). We identified several homologs of *Fol SIX* genes in all the legume-infecting ff. spp. The *Foc-*38-1 assembly contained homologs of *SIX5, 8, 11, 13* and *14*, whilst *Fop*-37622 contained *SIX1, 9, 13* and *14* (Table 6). All of the *SIX* homologs were encoded on scaffolds predicted to be dispensable except *Foc-38-1_SIX8* which is encoded on a scaffold with similarity to *Fol* core chromosome 5. There are many *SIX8* genes in *Fol* but none occur on core chromosome 5 (Table 6) [18]. While it is possible the *Foc*-38*-*1*-SIX8 -*encoding genomic region was mis-assembled, self-alignment of Illumina generated genomic read data supports the current assembly. There is the potential for transposon-mediated translocation of genes from dispensable regions into the core genome to have occurred. Thus it may be that the location of this gene has been shuffled in *Foc*-38-1, facilitated by the adjacent *mimps* and other TEs (*Foc-SIX8* is located on the end of Scaffold 138 next to a ‘*RESTLESS*’-like transposase).

**Table 6:** *SIX* gene presence on chromosomes/scaffolds in published *Fusarium* species^a^

	*F. oxysporum* f. sp. *lycopersici*	*F. oxysprum* f. sp. *medicaginis Fom*-5190a	*F. oxysporum* f. sp. *ciceris* 38-1	*F. oxysporum* f. sp. *pisi* 37622^b^	*F. oxysporum* f. sp. *brassica* Fo5176	*F. oxysporum* f. sp. *cubense* II5	*F. oxysporum* f. sp. *melonis* 26406_1	*F. solani (N. haematococca)*	*F. virguliforme* Mont-1	*F. graminearum* 1-4	*F. verticillioides*	*F. fujikuroi IMI 58289*
six1	14 (2nd half dup. on chr 14)	split over 696 and 1167 (partial dup. 241)	-	1.281, 1.315	contig00620	1.92, 1.101	1.500	-	-	-	-	-
six2	14	-	-	-	-	1.95	-	-	-	-	5	06
six3	14	-	-	-	-	-	-	-	-	-	-	-
six4	-	-	-	-	contig01435	1.102	-	-	-	-	-	-
six4b	_	-	-	-	contig04359	_	-	-	-	-	-	-
six5	14 (partial match Chr 5)	-	1215	partial match 1.4	-	-	1.4	-	-	-	-	-
six6	14	-	-	-	-	1.102	1.226	-	-	-	-	-
six7	14	-	-	-	-	-	-	-	-	-	-	-
six8	2, 3 (3 copies), 6 (2 copies), 7, 14 (2 copies)	420	0138	-	contig03501	1.127	-	-	-	-	-	-
six9	14	270	-	1.43	contig02779	1.86, 1.70	-	-	-	-	-	-
six10	14	-	-	-	-	-	-	-	-	-	-	-
six11	14	-	1126	-	-	-	1.532	-	-	-	-	-
six12	14	-	-	-	-	-	-	-	-	-	-	-
six13	6 (2 copies)	306 (split), 3812, 306	1143	1.90	-	1.216	1.222	-	-	-	-	-
six14	14 (1st half dup.)	-	0905	1.191	-	-	-	-	-	-	2	-

^a^ID of scaffold or contig showing TBLASTN matches with an Expect (E) value score below 10^−5^, *dup* duplicate. ^b^ *Fop*-37622 contained annotated homologs of SIX1 (FOVG_19815, FOVG_19730 and SIX9 (FOVG_17008) and matches by TBLASTN to unannotated versions of SIX13 and SIX14 which were manually annotated for this study (Additional file 23) and SIX5 where the coding sequence was interrupted by stop codons

Interestingly, only *SIX13* was found in all the legume infecting ff. spp. and this *SIX* gene is the only one in *Fol* (race 2) not found on CDC 14, but instead on CDC 6 [18]. As orthologs of this protein were also detected in ff. spp. infecting melons and banana it appears unlikely to have a role in legume host-specificity but may play a role in pathogenicity. We also observed *SIX* gene homologs in two other *Fusarium* species, *F. verticilloides* (*SIX2* and *SIX14*) and *F. fujikuroi* (*SIX2*) [85] (Table 6). The presence of *SIX* genes outside the species *F. oxysporum* has previously been observed in *F. verticilloides* (*SIX2*) [9] and *F. foetens* (*SIX1*) [62]. SIX1, SIX8, SIX9 and SIX13 homologs were identified in at least 5 out of the 7 *F. oxysporum* species analysed in Table 6, suggesting these proteins may play conserved roles in pathogenicity but not host-specificity, unless small amino acid changes govern their interaction with host proteins. Top BLASTP matches for SIX protein homologs in *Foc-*38-1 and *Fop-*37622 show that SIX8 is highly conserved between *Fom*-5190a and *Foc*-38-1, SIX13 between all three legume-infecting ff. spp. and SIX14 between pea- and chickpea-infecting isolates (Additional files 22 and 23). Phylogenetic relationships between the *SIX* genes encoded by *Fom*-5190a that were also present in other ff. spp. (Additional file 24) suggests that the relationship between proteins encoded on predicted dispensable scaffolds differs from that of the conserved core proteins (Additional file 10). This is not unexpected if dispensable genomic regions are indeed readily exchanged amongst different isolates [9], whilst core regions remain relatively stable or if gene content of dispensable regions is undergoing more frequent mutation and rearrangement facilitated by repetitive elements. This finding is supported by a recent study [80] that showed incongruent phylogenies between *SIX* genes (*1* and *6*) and the house-keeping gene *EF-1α* and additionally presented evidence of potential vertical transmission of *SIX6* between related isolates. It is most likely that the *SIX* genes have a common ancestry either laterally or vertically and we can speculate that minor sequence differences contributing to their alternate phylogeny may be the result of host-driven selection.

A recent study examining the landscape of the *Fol* pathogenicity chromosomes identified small clusters of *SIX* genes which were associated with a class of DNA transposons known as MITEs (Miniature Inverted-repeat Transposable Elements) [18]. These MITEs, include an upstream (within 1500 bp) incomplete fragment of the *Impala* transposon sequence (*miniature Impala* or ‘*mimp*’) [18] and often an additional downstream miniature *Fot5* transposon (*mFot5*) [18]. Their presence in gene-flanking sequences has also been used as a criterion to support the prediction of effector genes in ff. spp. infecting tomato and melons [10, 18]. We therefore searched for the presence of these TEs or their inverted repeats around the predicted legume-infecting ff. spp. *SIX* genes and effector candidates. The *SIX13* homolog residing on *Fom*-5190a Scaffold 306 is flanked by a partial *Impala* 430 bp upstream. In the *Foc*-38-1 assembly several *SIX* gene homologs had matches to *mimps* upstream (*SIX5, SIX8* and *SIX14*), and *SIX13* was flanked by a downstream complete *Fot5* transposase which may facilitate movement of this gene. In the case of *Foc*-38-1_*SIX13* and the *Fom*-5190a *SIX* genes where *mimps* were not identified upstream, the *SIX* gene homologs resided on short-length scaffolds with little surrounding sequence assembled, inhibiting the search for upstream or flanking intergenic sequences. In *Fop-*37622 upstream *mimps* were only identified close to *SIX1* (*FOVG_19815*)*, SIX9* and *SIX13* although the region upstream of *Fop-37622 SIX14* had undefined sequence hampering identification of a possible *mimp*.

### qRT-PCR validation of *Fom*-5190a effector candidates during host infection

**Fig. 2:**
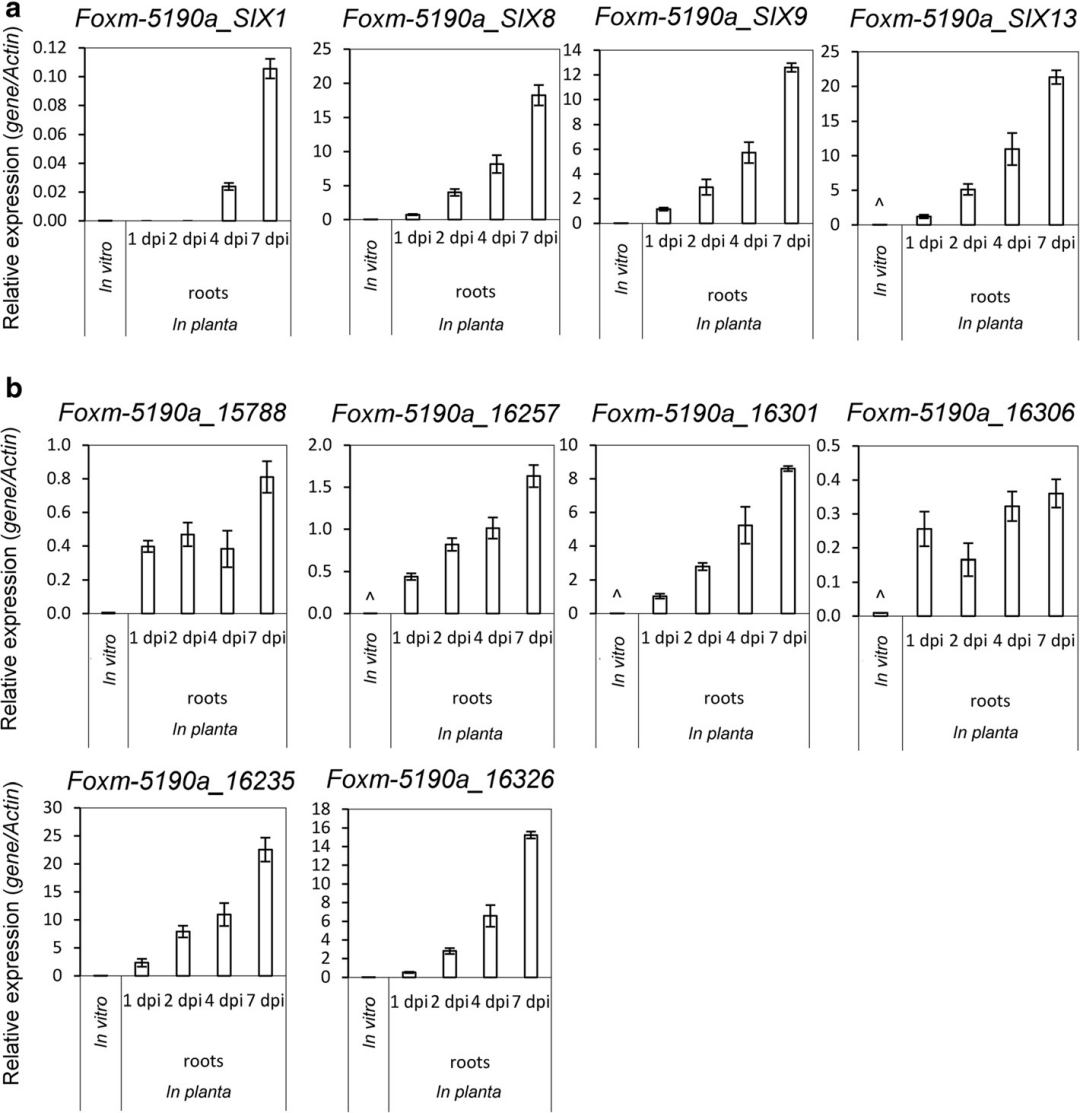
Expression of candidate pathogenicity genes *in vitro* versus *in planta*. **a** Expression of *SIX* genes and **b** candidate pathogenicity genes as determined by qRT-PCR in *in vitro* samples and *M. truncatula* DZA315 root samples harvested at 1, 2, 4 and 7 days post inoculation (dpi) with *Fom*-5190a. *In vitro* samples are averages ± SE of 3 biological replicates. *In planta* samples are averages ± SE of 4 biological replicates each consisting of pools of 10 seedlings. Gene expression levels are relative to the fungal actin gene (*FOXM-5190a_13365*). Note: ^ No detectable expression in 1 or 2 out of the 3 *in vitro* replicates

To validate our RNAseq data and determine expression of the ten shortlisted effector candidates over a longer period of infection, we examined via qRT-PCR their expression in vitro and over a 1–7 day *in planta* time-course in susceptible *M. truncatula* plants (Fig. 2). By 10 dpi, most infected plants had visible wilting symptoms and the majority of infected plants were killed by 21 dpi (Additional file 25a). Increasing fungal biomass over the course of infection was indicated by an increase in the amount of fungal ITS relative to plant ITS detected via qRT-PCR as the infection progressed (Additional file 25b).

Many fungal effector proteins are only expressed *in planta* and cannot be detected in vitro, or if so, at very low levels [57, 86] (reviewed in [87]). All of the *Fom*-5190a *SIX* gene homologs were expressed *in planta* and showed lower or no expression in vitro, exhibiting a pattern of increased expression over the course of the infection (1–7 days, Fig. 2a), peaking at 7 dpi with fold-inductions over in vitro ranging from 1050 to over 60,000. The other six predicted effector genes prioritised for initial follow up studies in *Fom*-5190a shared this expression pattern, albeit to various levels of induction (Fig. 2b). After *FOXM-5190a-SIX13*, *FOXM-5190a-16235* and *FOXM-5190a-16326* showed the largest fold-inductions in expression *in planta* versus in vitro, and both of their encoded proteins exhibited similar levels of identity across several *F. oxysporum* ff. spp. (Additional file 22). The *Fom*_5190a lineage specific gene *FOXM*_*5190a_16257*, showed strong expression *in planta* increasing over the course of infection but no or very little expression in vitro. This protein had an upstream *mimp* and no similarity to any proteins in the non-redundant database at NCBI (threshold e ≤ 1×10^−5^) making it a strong candidate for a host specific effector. However a Hidden Markov Model analysis identified a 38 aa region within the 91 aa protein sharing 58 % identity to a region from a hypothetical *Colletotrichum orbiculare* MAFF 240422 protein (Cob_00676) and other pathogenic fungi from the Ascomycota such as *Pseudocercospora fijiensis, Claviceps purpurea* and *Sphaerulina musiva*. An iterative search [88] of these sequences for distinct regions of similarity identified a motif resembling a zinc finger domain, suggesting FOXM-5190a_16257 may target host DNA sequences. The hypothetical FOXM_5190a_16306 protein which shares 99 % amino acid identity to FOC38_16051 had 27-fold up-regulated expression by 1 dpi, compared to in vitro*,* and showed a slight further increase in expression over the sampled time-course. Another candidate, *FOXM_5190a_16301,* whose product shares 95 % aa identity with a *Fop_*37622 protein (FOVG_19456) encodes a LysM domain and has some similarity to Ecp7 - a small, cysteine-rich, secreted effector protein identified in *Cladosporium fulvum* (syn. *Passalora fulva*) of unknown function [89] - which has homologs in several fungal species. This protein is an ortholog of a recently identified *F. oxysporum* f. sp. *melonis* candidate effector (FOM_19260), which has an upstream *mimp* and shares a promoter with another candidate effector [10]. However in *Fom*-5190 the lack of upstream assembled sequence meant we were unable to identify a *mimp* or potentially co-regulated gene. These genes and the others identified from the RNA expression analysis have been prioritised for further investigation of their roles in pathogenicity and host specificity.

## Conclusions

The addition of the genomic sequences of the legume-infecting *F. oxysporum* ff. spp*.* presented here adds to the accumulated bioinformatics resources for *Fusarium oxysporum formae speciales* and helps provide a powerful knowledge-base for predicting lineage-specific genes involved in host-specific pathogenicity. Analysis of pathogenicity-related CDC gene content conserved amongst the legume-infecting *Fusarium oxysporum* ff. spp. identified several *Fom*-5190a scaffolds enriched in genes with known or potential roles in pathogenicity, in particular carbohydrate active enzymes, cytochrome p450s, MFS and ABC transporters, fungal transcription factors as well as newly predicted effectors. While the source of these conserved gene sequences is yet to be elucidated, it is evident that parts of the potential dispensable chromosomes greater than just the repetitive regions are shuffled around within the genome of each f. sp., with those changes at least in *Fom*-5190a possibly associated with active transposable elements. As transposons can be a major source of genetic recombination in an asexual species, this may have contributed to the increased assembly size of *Foc-*38-1 and potentially the evolution of a large number of races in this f. sp.. Interestingly, the predicted dispensable scaffolds in *Foc*-38-1 and *Fom*-5190a shared more identity with *Fop*-37622 than with each other, and little with another legume-infecting *Fusarium* species (*F. solani*). They also shared to a similar degree, CDC content with ff. spp. that are pathogenic on non-legume plant species including *Arabidopsis* and melon, but not with *Fol*.

Combining this observation with the differing presence of *SIX* gene homologs across these ff. spp., points towards different sources for their origins of pathogenicity and suggests that pathogenicity on legumes is a complex phenotype. It is apparent that legume-pathogenicity is not simply governed by a small set of conserved genes retained from an ancestral species that are specific to legume-infecting isolates. It is possible, though unlikely based on our analyses, that the respective legume host-specific pathogenicity genes of the three legume-infecting ff. spp. may have the same origin but have since diverged significantly. Previous studies have shown that there can be multiple origins for pathogenicity on a given host within a *F. oxysporum* f. sp. and individual isolates phenotypically classified as a particular f. sp. can be more closely related to isolates belonging to a different f. sp. [90, 91]. We speculate that the origin of legume host-specific pathogenicity is not likely to have arisen from recent horizontal transfer events, as this would have resulted in greater sequence similarities than we observed. Whether the pathogenicity components were transferred as a whole chromosome from other *F. oxysporum* ff. spp. and subsequently reshuffled, mutated or partially lost remains to be elucidated, but will perhaps be revealed with the sequencing of additional legume-infecting ff. spp. and races.

For the model legume-infecting f. sp. *Fom*-5190a, we shortlisted a set of candidate effectors four of which showed greatest similarity to proteins from another legume pathogenic ff. spp., suggestive of a conserved role in legume pathogenicity. Initial verification via expression analysis of these candidates supports this approach and lays the framework that will facilitate functional characterisation of these candidates in subsequent studies, for the ultimate application of this knowledge towards the development of *Fusarium* wilt resistance in economically important legume crops.

## Methods

### Isolate sources

*F. oxysporum* f. sp. *medicaginis* (Weimer) W.C. Snyder & H.N. Hansen, (*Fom*-5190a, BRIP 5190a/IMI 172838, collection number 19911) was isolated from wilting leaves in a commercial field of *Medicago sativa* by John. A. Irwin in Boonah (QLD, Australia) in 1973 and is not known to infect other legume species. *F. oxysporum* Schlecht.: Fr. f. sp. *ciceris* (Padwick) Matuo and K. Sato (*Foc*-38-1), represents the most virulent race of this *forma specialis* (race 1) and was isolated from *Cicer arietinum* (chickpea) in Patancheru (Hyderabad, India). *F. oxysporum* f. sp. *pisi* (*Fop*-37622) was obtained from J.M. Kraft (USDA-ARS, Prosser, Washington, USA) via Hans VanEtten. It was determined by Dr. Kraft to be race 5.

### *Fusarium* growth conditions and DNA extraction

*Foc*-38-1 and *Fom*-5190a DNA extraction was performed using a cetyltrimethylammonium bromide (CTAB) based method as per Gao et al. [92]. *Foc*-38-1 was grown in potato dextrose broth in 250 ml flasks and incubated in a rotary shaker at 120 rpm at 25 °C. *Fom*-5190a was grown in a petri dish containing one-half-strength potato dextrose broth for 7 days at 22 °C. Mycelia were harvested by filtering through Miracloth, and washed repeatedly with sterile distilled water to remove excess of salts adhering to it. One gram of mycelium was crushed in liquid nitrogen prior to DNA extraction.

### Genome assembly

*Fom*-5190a and *Foc*-38-1 draft genome assemblies were assembled from paired-end and mate-paired Illumina 100 bp reads (Additional file 3). For *Fom*-5190a and *Foc*-38-1 assemblies, paired-end Illumina reads were trimmed of contaminating adapter sequences using Cutadapt 1.1 [93]. Reads less than 25 bp in length, after trimming, were discarded. Overlapping reads were merged using Flash 1.2.2 [94]. *Fom*-5190a 454 reads were trimmed/filtered using Mothur [95] to assess quality, remove homopolymers and convert raw SFF data to fasta and qual formats. Custom perl scripts were used to recognise titanium linkers and split sff reads into paired fastq format. For each isolate an initial assembly was created using Soapdenovo v2.04 [96] utilising merged and paired-end reads at the optimised kmer length of 27 for *Fom*-5190a and 19 for *Foc*-38-1. The resultant assemblies (derived from paired-end data only) were further scaffolded with both paired-end and mate-paired libraries, progressing from shortest to largest insert size using 5 successive rounds of SSPACE 2.0 [97] per mate-pair library. For *Fom*-5190a, 454 reads were incorporated following the 3 kb mate-pairs. Between iterations of SSPACE, scaffold gaps were filled by performing 5 rounds of GAPCLOSER (soapdenovo/1.05-gc1.12) [96]. Scaffolds obtained after all scaffolding and gap-closing was completed were filtered to remove those less than 200 bp for *Fom*-5190a and the assembly sequences were re-ordered by decreasing length. For SSPACE scaffolding of *Foc*-38-1 a minimum scaffold size of 1000 bp was used to eliminate potential assembly errors due to ‘shadow-library’ contamination (unfiltered paired-end fragments) in the Illumina mate-paired library. The completeness of the *Fom*-5190a and *Foc*-38-1 genome assemblies and their representation of their respective gene contents was assessed with CEGMA v 2.4 [46]. The whole-genome sequence of *Fop*-37622 was assembled using ALLPATHS-LG (version R37753) run with default parameters (kmer size of 96) [98]. Mitochondrial sequences were removed by searching against an NCBI mitochondrial database. *Ab initio* gene models were created combining predictions from GeneMarkES [99], GeneId [100], Augustus [101], GlimmerHMM [102] and SNAP [103], in conjunction with strand-specific PASA alignment [104] and GeneWise features from BLAST [105] against the UniRef90 database [106]. The gene models were further updated with RNAseq datasets. The resulting annotation was filtered to remove spurious genes that overlap with transposons [107].

### Sequence conservation analysis

Chromosome sequences of *F. oxysporum* f. sp. *lycopersici* (*Fol*) [GenBank: CM000589-603] [9] and *F. solani* (syn. *Nectria haematococca*) [11] (constructed from [GenBank: GG698896-GG699104], ordered and joined by 100 bp of “N” bases), which have been previously characterised into core and accessory chromosomes, were masked for *de novo*-predicted repetitive DNA sequences using RepeatMasker v4.0.5 [108]. Repeat-masked *Fol* and *F. solani* chromosome sequences were then compared to the genome assembly sequences of other published/publicly available *Fusarium* spp. via MUMmer v3.1 (PROmer --mum, delta-filter) [55]. The percentage of the un-masked length of each chromosome that was covered by one or more PROmer matches was compared to high-quality reference sequences in which CDCs have been well defined - *Fol* and *F. solani* - via BEDTools CoverageBed [109] and visualised using Circos v0.67-1 [110] (Fig. 1 and Additional file 9).

### Prediction of non-core scaffolds

Scaffolds representing *Fom*-5190a*, Fop*-37622 and *Foc*-38-1 dispensable chromosomes were predicted based on MUMmer v3.0 (promer --mum, delta-filter -g) [55] alignments to repeat-masked sequences of *Fol* and *F. solani* of which genome assemblies for both species contain full length chromosome sequences that have been previously characterized as core and accessory chromosomes [9]. *Foc*-38-1, *Fop*-37622 and *Fom*-5190a scaffolds with > = 30 % of their length covered by unique promer matches to core chromosomes of *Fol* and *F. solani* (i.e. excluding *Fol* CDCs 3, 6, 14 and 15 [9] or *F. solani* CDCs 14, 15 and 17 [11]) were assigned as core scaffolds and all others were considered lineage specific and thus potentially part of a CDC.

### Annotation of genome features

Protein-coding gene regions of *Fom*-5190a and *Foc*-38-1 were initially predicted *de novo* via GeneMark-ES v 20120203 [99] using a minimum contig length of 200 bp. Protein sequences from publicly available *Fusarium* spp. genome projects, PHI-base [111, 112] and secreted-in-xylem (SIX) protein sequences obtained from GenBank were used to refine the GeneMark-ES predicted annotations via EVidenceModeller v 2012-06-25 [113]. Regions of both assemblies homologous to SIX proteins by TBLASTN (e-value threshold 1e-^5^) [105] were manually annotated based on homology and RNAseq data if not previously predicted.

In order to ensure that genes that played potential roles in pathogenicity and host specificity were correctly annotated following automated gene annotation, the assemblies were examined for matches to genes known to be involved in pathogenicity in other fungi [18, 84, 111, 112, 114]. Genes of interest that had not been annotated correctly were manually annotated based on homology and the RNA-seq data.

Repetitive DNA regions were predicted within genome assemblies of *Fom*-5190a, *Foc*-38-1 and other publicly available *Fusarium* spp. (Additional file 8) by both *de novo* prediction and comparison with the RepBase database of known fungal repeat sequences [115]. Repeat families were predicted *de novo* using RepeatScout v1.0.5 [116] (default parameters), the outputs of which were clustered for redundant or fragmented repeat families via Cap3 (-h 70 -z 1 -p 70) [117]. The non-redundant *de novo* repeat families were mapped to genome assemblies via RepeatMasker v4.0.5 [108] (crossmatch, -no_is -s) to determine the novel repetitive DNA content of each genome. To estimate the relative proportions of known transposon classes and sub-classes, each *Fusarium* genome sequence was also searched via RepeatMasker v4.0.5 (parameters: -no_is -qq) for matches to RepBase v20140131 [115].

The genome assemblies of *Fom*-5190a, *Foc*-38-1 and other publicly-available *Fusarium* spp. were also searched for non-coding RNA (ncRNA) using the cmsearch program from infernal 1.1rc4 (search mode) [118] using the Rfam 11.0 database [119–121]. Additionally, ribosomal RNA (rRNA) regions were predicted using RNAmmer 1.2 [122] and transfer RNA (tRNA) genes were predicted using tRNAscan v 1.3 [123].

### Annotation of protein functions

Within the protein translations of gene annotations of *Fom*-5190a, *Fop-*37622 and *Foc*-38-1, conserved amino-acid domains were identified using HMMER v 3.0 [124], against the PFAM-A database (v 27.0) (gathering cut-offs) and InterProScan v4.8 [125, 126]. Carbohydrate-active enzyme (CAZyme) (www.cazy.org) [67, 68] annotations were assigned to protein sequences via dbCAN [127] and HMMER v3.0 [124] with default settings. BLAST (version 2.2.26) [105] searches were performed at a significance score threshold of 1e^−5^ unless otherwise specified.

Orthologs of genes known to be involved in pathogenicity in other species (PHIbase) or *F. oxysporum* f. sp. (SIX genes) were identified via reciprocal BLAST analysis of both the predicted proteins and the scaffolds (1e^−5^) Predicted *Fom*-5190a and *Foc*-38-1 proteins and genomic scaffolds were also compared to 2309 protein sequences from the Pathogen Host Interaction database (PHIbase- version 3.5) [111, 112] which have been experimentally tested for roles in pathogenicity. Matches were considered only for reciprocal BLAST matches below an expectation value of 1×10^−5^. Genes potentially involved in the synthesis of secondary metabolites were identified using SMURF [128]. The potential localisation of predicted proteins was analysed via WoLFP SORT [129] and Phobius [130].

The proteins of the five reference *Fusarium* genomes (*Fo5176*, *Fol*, *F. gaminearum, F. solani, F. verticilloides*) and those of *Fom*-5190a, *Foc*-38-1 and *Fop-*37622 were classified for the purpose of this study as small secreted proteins (SSPs), based on criteria previously used by Ohm and colleagues [131]. These criteria include prediction of secretion by SignalP v.4.1b [132], with one or less N terminal transmembrane domains as predicted by TMHMM v. 2.0c [133] although the length cut-off was increased from 200 to 300 aa to include known effector proteins identified in *Fusarium* sp. such as the SIX proteins.

### Statistical examination for over- or under-representation of protein functional attributes

The number of genes with specific functional attributes (Pfam domains [65]) was compared between predicted core and dispensable scaffolds and compared using Fisher’s exact test. Those that were increased on dispensable scaffolds with a significance threshold of *p ≤* 0.05 are listed in Additional files 15, 16 and 17.

### Orthology

Proteinortho v4.26 [134] was used to detect orthologs of *Fom*-5190a, *Fop-*37622 and *Foc*-38-1 compared with 41 isolates of the following fungal species: *Alternaria brassicicola, Ashbya gossypii, Blumeria gramminis* f. *hordei, Botrytis cinerea, Cladosporium fulvum (syn. Passalora fulva), Coccidiodes immitis, Fusarium acuminatum, Fusarium culmorum, Fusarium graminearum, Fusarium fujikuroi, Fusarium incarnartum-Fusarium equiseti* species complex*, Fusarium oxysporum, Fusarium oxysporum* f. *sp. conglutinans, Fusarium oxysporum* f. sp. *cubense, Fusarium oxysporum* f. sp. *lycopersici, Fusarium oxysporum* f. sp*. melonis, Fusarium oxysporum* f. sp. *pisi, Fusarium oxysporum* f. sp*. radicis-lycopersici, Fusarium oxysporum* f. sp. *raphani, Fusarium oxysporum* f. sp. *vasinfectum, Fusarium pseudograminearum, Fusarium solani* (*syn. Nectria haematococca*)*, Fusarium verticilliodes* (*syn. Gibberella fujikuroi*), *Grosmania clavigera, Leptosphaeria maculans, Magnaporthe oryzae* (*syn. grisea*), *Mycosphaerella graminicola (syn. Zymoseptoria tritici), Neurospora crassa, Parastagonospora nodorum, Podospora anserina, Saccharomyces cerevisiae, Sordaria macrospora, Trichoderma reesei, Tuber melanosporum, Uncinocarpus reesii, Verticillium dahliae.* Details of the specific isolates and data sets used are provided in Additional file 5. Orthologs were determined via reciprocal BLASTP using parameters: −e = 1e^−5^, alg.conn. = 0.1, coverage = 0.5, percent_identity = 25, adaptive_similarity = 0.75, retaining both pairs and singletons (Additional file 6). These isolates were selected based on their close relation to the genus *Fusarium* or the fact that they either shared a similar host range or infection mode or had a very diverse one. *F. virguliforme* and *F. circinatum* were used for comparison at the whole genome sequence level only.

### Phylogeny

From the Proteinortho predictions, 100 proteins were randomly chosen that had predicted one-to-one orthologies across all *Fusarium* sp. genome assemblies and protein sequences were concatenated, all of the orthologs in f. sp. *medicaginis* were encoded on predicted core scaffolds. Multiple sequence alignments were calculated using Clustal Omega version 1.2.1 [135] and phylogenetic trees constructed using RAxML version 8.1.20 [136]. Workflows were automated using the ete build function of the ETE toolkit [137] and trees were drawn using ete view with branch support values shown.

### Sample preparation for RNA-seq and qRT-PCR

For *F. oxysporum* inoculations of *M. truncatula* the isolate *Fom*-5190a was maintained on sterile filter paper and grown on one-half-strength potato dextrose agar. For spore production, 3 agar plugs were removed to inoculate flasks containing 100 mL of one-half-strength potato dextrose broth and grown for 3 days at 28 °C/100 rpm. The inoculum was drained through Miracloth (Calbiochem, San Diego), centrifuged to pellet spores, and resuspended in sterile distilled water before quantification with a haemocytometer. The spore concentration was adjusted to 1 × 10^6^ spores mL^−1^ in sterile distilled water and used for plant inoculations. The *M. truncatula* accession DZA315.15 susceptible to *Fom*-5190a (J. Lichtenzveig unpublished, this work) was germinated on damp filter paper, transplanted into 30 mm Jiffy-7 peat pellets and grown under a short-day light regime (8-h light/16-h dark) in a growth room set at 22 °C. After 2 weeks, roots protruding from the peat pellets were removed. Peat pellets were then inoculated by placing them in a petri dish of spore suspension for 5 min, followed by a further 1 mL of spore suspension added to the base of the hypocotyl. Inoculated pellets were transferred to growth trays lined with a plastic sheet and a thin layer of damp vermiculite, covered with a clear plastic dome to maintain humidity, and incubated under a long-daylight regime (16-h light/8-h dark) growth room set at 28 °C.

### RNA isolation

For qRT-PCR and RNA-seq experiments on *Fom*-5190a infected *M. truncatula* accession DZA315.15, root tissue was collected from 10 plants per replicate at 1, 2, 4 and 7 days post inoculation and pooled for RNA extraction. For *Fom*-5190a *in vitro* samples, mycelia was grown in a petri dish containing one-half-strength potato dextrose broth for 7 days at 22 °C and mycelia harvested by filtering through Miracloth. Three or four separate biological replicates were taken for all experiments, then frozen in liquid nitrogen, and stored at −80 °C. RNA extraction was performed using a Trizol extraction followed by DNase treatment using TURBO DNase (Ambion). RNA samples were cleaned via RNeasy mini spin columns (Qiagen).

### qRT-PCR

Following RNA isolation and DNase treatment, complementary DNA synthesis was performed using SuperscriptIII reverse transcriptase (Invitrogen) with oligo (dT) (Invitrogen) and RNasin (Promega) with 1 μg of input RNA. qRT-PCR was performed using SsoFast EvaGreen Supermix (Bio-Rad) on a CFX384 (Bio-Rad) system. Thermoycling and melt-curve conditions were as described by Oñate-Sánchez et al. [138]. Absolute gene expression levels relative to *F. oxysporum* actin were used for each complementary DNA sample using the equation: relative ratio gene of interest/*Actin* = (E*gene*^-Ct *gene*^)/(E*Actin*^-Ct *Actin*^) where Ct is the cycle threshold value. *Medicago* root samples were verified for even abundance of plant input material using the *M. truncatula B-tubulin* reference gene [139] which was found to be within ± 1 Ct across all samples. Primer sequences are listed in Additional file 26.

### RNA-seq library construction, Illumina sequencing and read-mapping

RNA integrity was confirmed using the Agilent 2100 Bioanalyser Plant Nano system (Agilent Biotechnologies). Stranded Illumina TruSeq libraries were generated from 1 μg of total RNA and sequenced (100 bp paired end reads) on an Illumina HiSeq platform by the Australian Genome Research Facility (AGRF). 51–60 million reads were generated for each sample. RNA-seq paired-end reads were trimmed for low-quality base-calls and Illumina adapter sequences via Cutadapt (v1.1, parameters: −quality-cutoff 30 --overlap 10 --times 3 –minimum-length 25) [93]. Reads trimmed to less than 25 bp were discarded and remaining reads sorted into pairs and singleton reads. RNA-seq reads were mapped to the *Fom*-5190a genome assembly via Tophat2 (v2.0.9, parameters: −b2-very-sensitive -r 80 --mate-std-dev 40 -i 20 -I 4000 -g 20 --report-secondary-alignments --report-discordant-pair-alignments -m 0 --min-coverage-intron 20 --microexon-search --library-type fr-firststrand) [140].

### Data sources and acknowledgements

This research was undertaken with the assistance of resources from the Australian Genome Research Facility (AGRF) and the National Computational Infrastructure Specialised Facility for Bioinformatics (NCI-SF Bioinformatics), which are both supported by the Australian Government. The work was supported by iVEC through the use of advanced computing resources located at the Pawsey Supercomputing Centre. Data used for comparative analysis was obtained from NCBI, unpublished *Fusarium* data sets obtained from “*Fusarium* Comparative Sequencing Project, Broad Institute of Harvard and MIT (http://www.broadinstitute.org/)”, Broad Institute Genomics Platform, Feb 2014. *F. solani* and *F. fujikuoroi* data was obtained from the JGI Genome portal (http://genome.jgi.doe.gov/), all data sources are outlined in Additional file 4*.* We also thank Elaine Smith for excellent technical assistance and Dr Donald Gardiner for critical reading of the manuscript and useful suggestions.

### Availability of supporting data

Trimmed sequencing data for *Fom*-5190a was deposited into the NCBI/GenBank database under BioProject number PRJNA294248 (http://www.ncbi.nlm.nih.gov/bioproject/?term=PRJNA294248). This Whole Genome Shotgun project has been deposited at DDBJ/ENA/GenBank under the accession LSNI00000000. The version described in this paper is version LSNI01000000. Raw sequence data and the assembly for Foc-38-1 were deposited into the NCBI/GenBank database under BioProject numbers PRJNA282695 and PRJNA188291 (http://www.ncbi.nlm.nih.gov/bioproject/?term=282695, http://www.ncbi.nlm.nih.gov/bioproject/PRJNA188291). The genome and annotation of the *Fop-*37622 were deposited at DDBJ/EMBL/GenBank under the accession number AGBI00000000.1 (http://www.ncbi.nlm.nih.gov/nuccore/AGBI00000000).

## Additional files

Additional file 1:
**Summary of conditionally-dispensable chromosomes in Ascomycete plant pathogens.** (DOCX 36 kb) Additional file 2:
***F. oxysporum*** **f. sp.** ***medicaginis*** **(** ***Fom-*** **5190a) and** ***F. oxysporum*** **f. sp.** ***ciceris*** **(** ***Foc*** **-38-1) disease symptoms.** (a) *Fom-*5190a disease symptoms on *Medicago truncatula*. The left image shows the *Fusarium* wilt susceptible *M. truncatula* accession DZA315 at 10 days post inoculation (dpi) displaying wilting leaf symptoms. The centre image shows an uprooted plant from the same experiment at 18 dpi showing stunted, necrotic roots compared to a mock inoculated seedling (right image) at the same time-point. (b) *F. oxysporum* f. sp. *ciceris* (*Foc*-38-1) disease symptoms on *Cicer arietinum*. The left and centre images show the *Fusarium* wilt susceptible *C. arietinum* accession JG 62 at 9 and 18 days post inoculation respectively, with wilting leaf symptoms prominent. A mock/control inoculated seedling (right image) is shown as a reference. (TIF 8644 kb) Additional file 3:
**Sequencing data used for genome assemblies.** (DOCX 13 kb) Additional file 4:
**Protein/gene set comparisons across** ***Fusarium*** **sp.** (DOCX 11 kb) Additional file 5:
**Summary of genome and protein sequence resources used for analysis.** (XLSX 20 kb) Additional file 6:
**Orthology analysis.** (XLSX 23360 kb) Additional file 7:
**G:C content analysis by scaffold/chromosome for legume-infecting ff. spp., and** ***Fol*** **and** ***F. solani.*** (XLSX 338 kb) Additional file 8:
**Repeat analysis.** (XLSX 20 kb) Additional file 9:
***F. solani*** **chromosomes highlighting features of CDCs in comparison to core chromosomes.** Outer ring-*F. solani* chromosomes CDCs highlighted in red. Inner rings: (a) gene density in 100 kb windows, (b) repeat density in 100 kb windows, (c) GC content in 50 k bp windows range 45-55 %, (d) *Region of F. solani* chromosomes overlapped by *Fom-*5190a sequences, (e) *Foc-*38-1, (f) *F. oxysporum* f. sp. *pisi*-37622 HDV247, (g) *F. oxysporum* f. sp. *brassica Fo5176*, (h) *F. oxysporum* f. sp. *melonis,* (i) *F. oxysporum* f. sp. *lycopersici,* (j) *F. fujikuori,* (k) *F. verticilliodes,* (l) *F. virguliforme*, and (m) *F. graminearum*. (TIFF 1823 kb) Additional file 10:
***Fusarium*** **sequences conserved with core and dispensable chromosomes of** ***Fol*** **and** ***F_solani.*** (XLSX 40 kb) Additional file 11:
**Phylogenetic tree illustrating the relationship between the** ***Fusarium*** **sp. isolates used in this study Branch support values are shown in red.** This tree illustrates that the legume-infecting isolates do not appear to be more closely related to each other than to other ff. spp. (PDF 21 kb) Additional file 12:
**Ortholog found only in legume-infecting Fusarium sp.** (DOCX 11 kb) Additional file 13:
**Dispensable scaffold predictions in** ***Fom*** **-5190,** ***Foc*** **-38-1 and** ***Fop*** **-37622.** (XLS 2255 kb) Additional file 14:
**Comparison of differences in length between core and lineage specific scaffolds across** ***Fusarium*** **species.** (DOCX 12 kb) Additional file 15:
**Pfam domains more abundant on predicted dispensable scaffolds in** ***Fom*** **-5190a.** (DOCX 18 kb) Additional file 16:
**Pfam domains more abundant on predicted dispensable scaffolds in** ***Foc*** **-38-1.** (DOCX 14 kb) Additional file 17:
**Pfam domains more abundant on predicted dispensable scaffolds in** ***Fop*** **-37622.** (DOCX 16 kb) Additional file 18:
**Proteins with similarities to known fungal pathogenicity genes and their expression at 2 dpi in** ***Fom*** **-5190a.** (DOCX 16 kb) Additional file 19:
**Properties of predicted** ***Fom*** **-5190a genes/proteins.** (XLSX 5099 kb) Additional file 20:
**Properties of predicted** ***Foc*** **-38-1 genes/proteins.** (XLSX 6173 kb) Additional file 21:
**Properties of predicted** ***Fop-37622*** **genes/proteins.** (XLSX 4021 kb) Additional file 22:
**Best BLASTP matches of** ***Fom*** **-5190a candidate effector proteins and SIX protein orthologs versus NCBI non-redundant protein database (Feb 2015).** (DOCX 15 kb) Additional file 23:
**Best BLASTP matches of** ***Foc*** **38-1 and** ***Fop*** **-37622 SIX protein orthologs and predicted** ***Fop*** **_SIX13 and** ***[*** **_SIX14 aa sequences.** (DOCX 18 kb) Additional file 24:
**Phylogenetic trees illustrating the relationship between SIX proteins detected as encoded in** ***Fusarium oxysporum*** **f. sp.** ***medicaginis*** **and other ff. spp.** (a) SIX1, (b) SIX8, (c) SIX9 and (d) SIX13. The relationship between the SIX proteins suggests a greater similarity between those from the legume–infecting ff. spp. than the phylogenetic analysis based on core proteins identified. (PNG 165 kb) Additional file 25:
**Disease symptoms and relative abundance of** ***Fom*** **-5190a in infected** ***M. truncatula*** **DZA315 root samples.** (a) Disease symptoms of DZA315 plants at 14 days post treatment with *Fom*-5190a or a control (mock) treatment. (b) Relative *Fom*-5190a fungal abundance was determined by qRT-PCR expression of *Fom*-*5190a_18S* relative to *M. truncatula*_*18S* expression in *M. truncatula* DZA315 root samples harvested at 1, 2, 4 and 7 days post inoculation (dpi). Samples are averages ± SE of 4 biological replicates consisting of pools of 10 seedlings. (TIF 2196 kb) Additional file 26:
**Primer sequences used for qRT-PCR.** (DOCX 12 kb)
