# Supplementary material for: Comparative genomics and prediction of conditionally dispensable sequences in legume–infecting Fusarium oxysporum formae speciales facilitates identification of candidate effectors
Source: BMC Genomics. 2016 Mar 5;17:191. doi: 10.1186/s12864-016-2486-8 (PMC4779268; doi:10.1186/s12864-016-2486-8)
Supplement: Additional file 1: — Summary of conditionally-dispensable chromosomes in Ascomycete plant pathogens. (DOCX 36 kb) [file 12864_2016_2486_MOESM1_ESM.docx]

Additional File 1 Summary of conditionally-dispensable chromosomes in Ascomycete plant pathogens.

| **Species** | References |
| --- | --- |
| *Zymoseptoria tritici* | [[1](#_ENREF_1)] |
| *Leptosphaeria maculans* | [[2](#_ENREF_2), [3](#_ENREF_3)] |
| *Alternaria alternata* | [[4](#_ENREF_4), [5](#_ENREF_5)] |
| *Cochliobolus spp.* | [[6-9](#_ENREF_6)] |
| *Fusarium solani* (syn. *Nectria haematococca*) | [[10-18](#_ENREF_10)] |
| *Fusarium fujikuroi* | [[19](#_ENREF_19), [20](#_ENREF_20)] |
| *Fusarium oxysporum* f. sp. *lycopersici* | [[2](#_ENREF_22)1] |
| *Fusarium oxysporum* f. sp *cubense* | [[2](#_ENREF_23)2] |
| *Magnaporthe grisea* | [[2](#_ENREF_25)3] |

1. Goodwin SB, M'Barek S B, Dhillon B, Wittenberg AH, Crane CF, Hane JK, Foster AJ, Van der Lee TA, Grimwood J, Aerts A *et al*: **Finished genome of the fungal wheat pathogen *Mycosphaerella graminicola* reveals dispensome structure, chromosome plasticity, and stealth pathogenesis**. *PLoS genetics* 2011, **7**(6):e1002070.

2. Balesdent MH, Fudal I, Ollivier B, Bally P, Grandaubert J, Eber F, Chèvre AM, Leflon M, Rouxel T: **The dispensable chromosome of *Leptosphaeria maculans* shelters an effector gene conferring avirulence towards *Brassica rapa***. *New Phytologist* 2013, **198**(3):887-898.

3. Leclair S, Ansan-Melayah D, Rouxel T, Balesdent M-H: **Meiotic behaviour of the minichromosome in the phytopathogenic ascomycete *Leptosphaeria maculans***. *Current genetics* 1996, **30**(6):541-548.

4. Hatta R, Ito K, Hosaki Y, Tanaka T, Tanaka A, Yamamoto M, Akimitsu K, Tsuge T: **A conditionally dispensable chromosome controls host-specific pathogenicity in the fungal plant pathogen *Alternaria alternata***. *Genetics* 2002, **161**(1):59-70.

5. Akagi Y, Akamatsu H, Otani H, Kodama M: **Horizontal chromosome transfer, a mechanism for the evolution and differentiation of a plant-pathogenic fungus**. *Eukaryotic cell* 2009, **8**(11):1732-1738.

6. Ahn J-H, Walton JD: **Chromosomal organization of TOX2, a complex locus controlling host-selective toxin biosynthesis in *Cochliobolus carbonum***. *The Plant cell* 1996, **8**(5):887-897.

7. Masel A, He C, Poplawski AM, Irwin JA, Manners JM: **Molecular evidence for chromosome transfer between biotypes of *Colletotrichum gloeosporioides***. *MPMI-Molecular Plant Microbe Interactions* 1996, **9**(5):339-348.

8. Masel AM, Irwin JA, Manners JM: **DNA addition or deletion is associated with a major karyotype polymorphism in the fungal phytopathogen *Colletotrichum gloeosporioides***. *Molecular and General Genetics* 1993, **237**(1-2):73-80.

9. Tzeng T-H, Lyngholm L, Ford C, Bronson C: **A restriction fragment length polymorphism map and electrophoretic karyotype of the fungal maize pathogen *Cochliobolus heterostrophus***. *Genetics* 1992, **130**(1):81-96.

10. Coleman JJ, Rounsley SD, Rodriguez-Carres M, Kuo A, Wasmann CC, Grimwood J, Schmutz J, Taga M, White GJ, Zhou S *et al*: **The genome of *Nectria haematococca*: contribution of supernumerary chromosomes to gene expansion**. *PLoS genetics* 2009, **5**(8):e1000618.

11. Covert SF, Enkerli J, Miao VP, VanEtten HD: **A gene for maackiain detoxification from a dispensable chromosome of *Nectria haematococca***. *Molecular and General Genetics MGG* 1996, **251**(4):397-406.

12. Funnell DL, VanEtten HD: **Pisatin demethylase genes are on dispensable chromosomes while genes for pathogenicity on carrot and ripe tomato are on other chromosomes in *Nectria haematococca***. *Molecular plant-microbe interactions* 2002, **15**(8):840-846.

13. Kistler HC, Benny U: **Autonomously replicating plasmids and chromosome rearrangement during transformation of *Nectria haematococca***. *Gene* 1992, **117**(1):81-89.

14. Kistler HC, Van Etten HD: **Three non-allelic genes for pisatin demethylation in the fungus *Nectria haematococca***. *Journal of general microbiology* 1984, **130**(10):2595-2603.

15. Mackintosh SF, Matthews DE, VanEtten HD: **Two additional genes for pisatin demethylation and their relationship to the pathogenicity of *Nectria haematococca* on pea**. *Mol Plant-Microbe Interact* 1989, **2**:354-362.

16. Miao VP, Covert SF, VanEtten HD: **A fungal gene for antibiotic resistance on a dispensable (" B") chromosome**. *Science* 1991, **254**(5039):1773-1776.

17. Miao VP, Matthews DE, VanEtten HD: **Identification and chromosomal locations of a family of cytochrome P-450 genes for pisatin detoxification in the fungus *Nectrla haematococca***. *Molecular and General Genetics MGG* 1991, **226**(1-2):214-223.

18. Wasmann C, VanEtten HD: **Transformation-mediated chromosome loss and disruption of a gene for pisatin demethylase decrease the virulence of *Nectria haematococca* on pea**. *MPMI-Molecular Plant Microbe Interactions* 1996, **9**(9):793-803.

19. Xu J-r, Leslie JF: **A genetic map of *Gibberella fujikuroi* mating population A (*Fusarium moniliforme*)**. *Genetics* 1996, **143**(1):175-189.

20. Xu J-R, Yan K, Dickman MB, Leslie JF: **Electrophoretic karyotypes distinguish the biological species of *Gibberella fujikuroi* (*Fusarium* section *Liseola*)**. *MPMI-Molecular Plant Microbe Interactions* 1995, **8**(1):74-84.

21. Ma LJ, van der Does HC, Borkovich KA, Coleman JJ, Daboussi MJ, Di Pietro A, Dufresne M, Freitag M, Grabherr M, Henrissat B *et al*: **Comparative genomics reveals mobile pathogenicity chromosomes in *Fusarium***. *Nature* 2010, **464**(7287):367-373.

22. Boehm E, Ploetz R, Kistler HC: **Statistical analysis of electrophoretic karyotype variation among vegetative compatibility groups of *Fusarium oxysporum* f. sp. *cubense***. *Molecular Plant Microbe Interactions* 1994, **7**(2):196-207.

23. Orbach MJ, Chumley FG, Valent B: **Electrophoretic karyotypes of *Magnaporthe grisea* pathogens of diverse grasses**. *MPMI-Molecular Plant Microbe Interactions* 1996, **9**(4):261-271.
