# Supplementary figures and images for: Comparative genomics and prediction of conditionally dispensable sequences in legume–infecting Fusarium oxysporum formae speciales facilitates identification of candidate effectors
Source: BMC Genomics. 2016 Mar 5;17:191. doi: 10.1186/s12864-016-2486-8 (PMC4779268; doi:10.1186/s12864-016-2486-8)

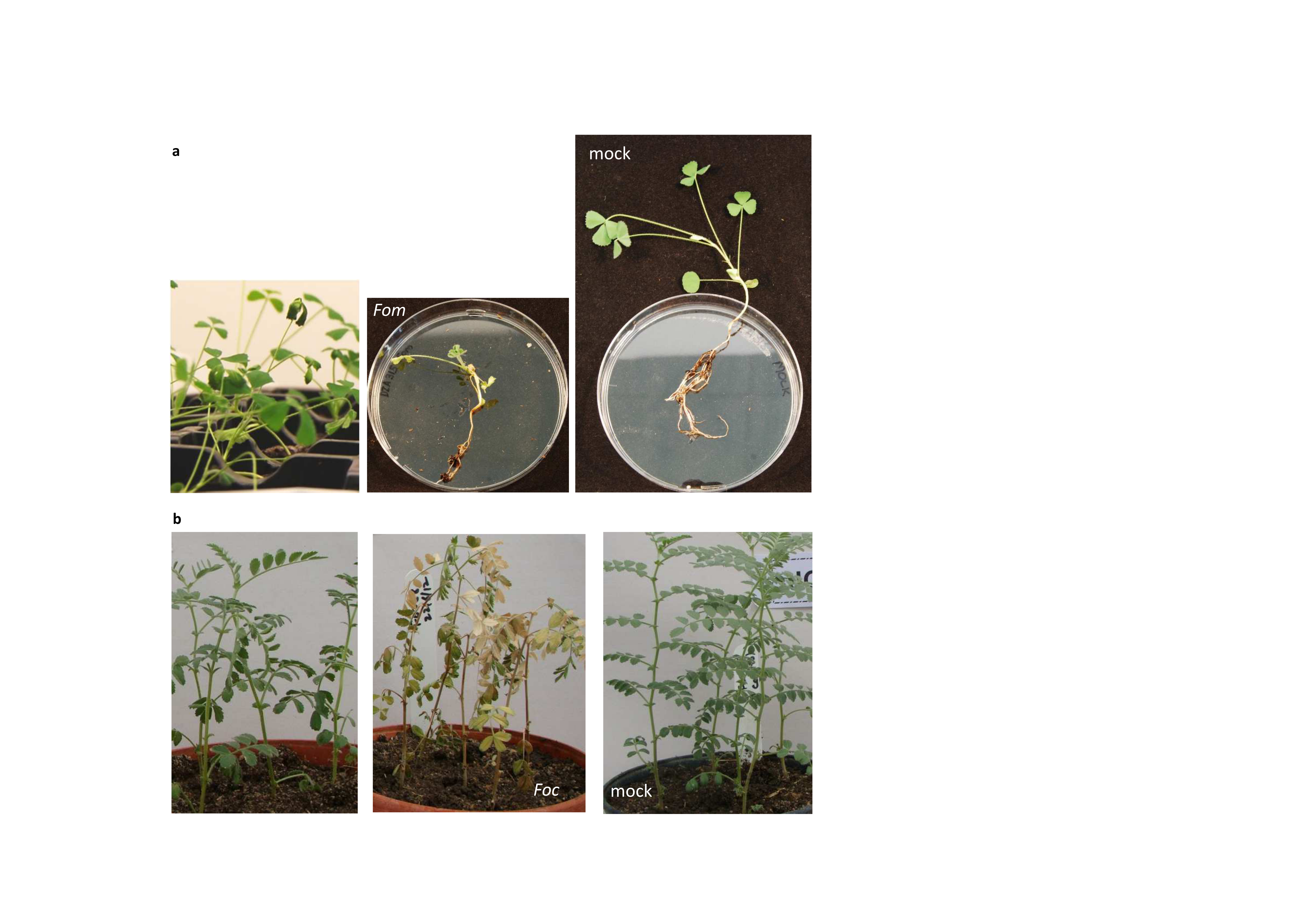

Supplement: Additional file 2: — F. oxysporum f. sp. medicaginis ( Fom- 5190a) and F. oxysporum f. sp. ciceris ( Foc -38-1) disease symptoms. (a) Fom-5190a disease symptoms on Medicago truncatula. The left image shows the Fusarium wilt susceptible M. truncatula accession DZA315 at 10 days post inoculation (dpi) displaying wilting leaf symptoms. The centre image shows an uprooted plant from the same experiment at 18 dpi showing stunted, necrotic roots compared to a mock inoculated seedling (right image) at the same time-point. (b) F. oxysporum f. sp. ciceris (Foc-38-1) disease symptoms on Cicer arietinum. The left and centre images show the Fusarium wilt susceptible C. arietinum accession JG 62 at 9 and 18 days post inoculation respectively, with wilting leaf symptoms prominent. A mock/control inoculated seedling (right image) is shown as a reference. (TIF 8644 kb) [file 12864_2016_2486_MOESM2_ESM.tif]

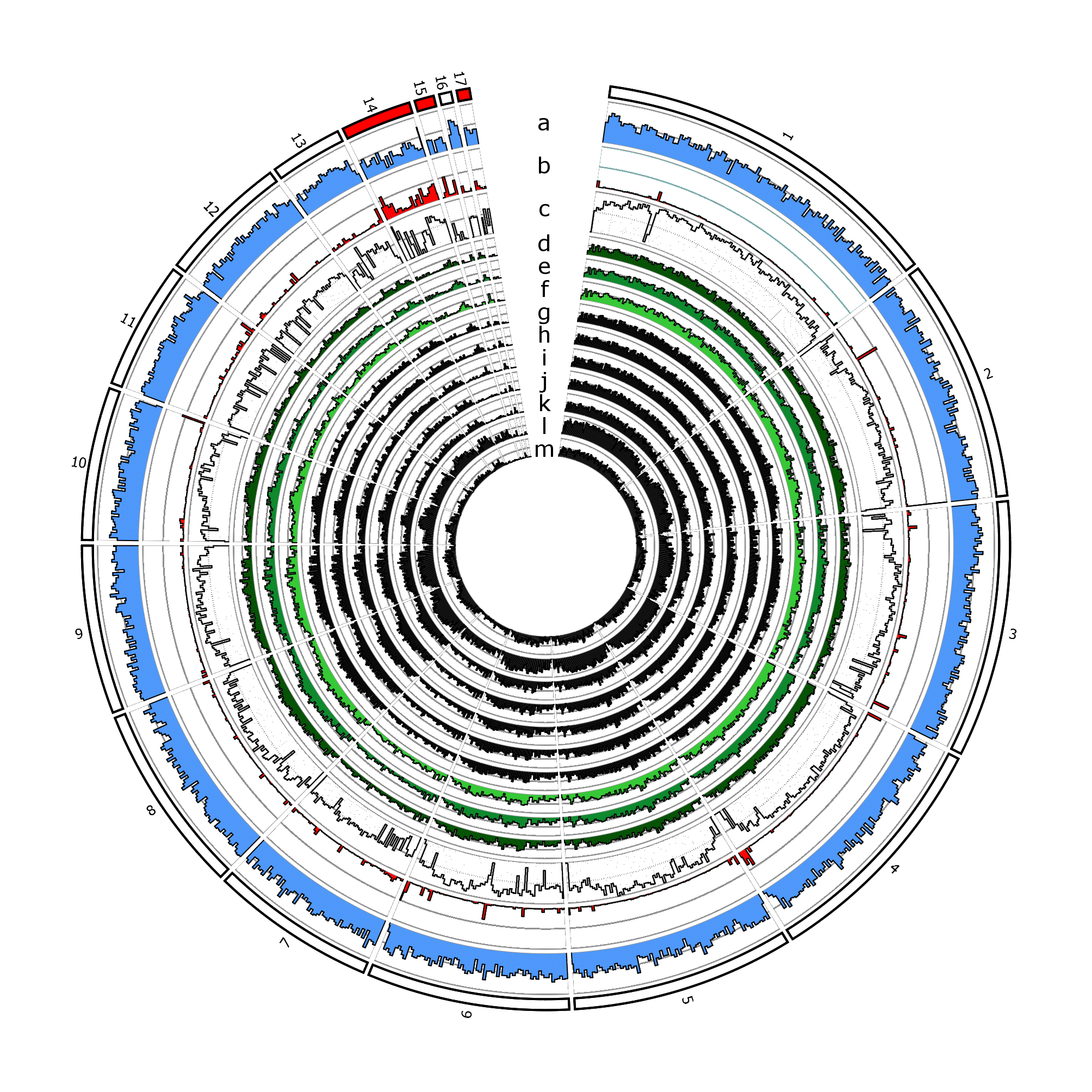

Supplement: Additional file 9: — F. solani chromosomes highlighting features of CDCs in comparison to core chromosomes. Outer ring-F. solani chromosomes CDCs highlighted in red. Inner rings: (a) gene density in 100 kb windows, (b) repeat density in 100 kb windows, (c) GC content in 50 k bp windows range 45-55 %, (d) Region of F. solani chromosomes overlapped by Fom-5190a sequences, (e) Foc-38-1, (f) F. oxysporum f. sp. pisi-37622 HDV247, (g) F. oxysporum f. sp. brassica Fo5176, (h) F. oxysporum f. sp. melonis, (i) F. oxysporum f. sp. lycopersici, (j) F. fujikuori, (k) F. verticilliodes, (l) F. virguliforme, and (m) F. graminearum. (TIFF 1823 kb) [file 12864_2016_2486_MOESM9_ESM.tiff]

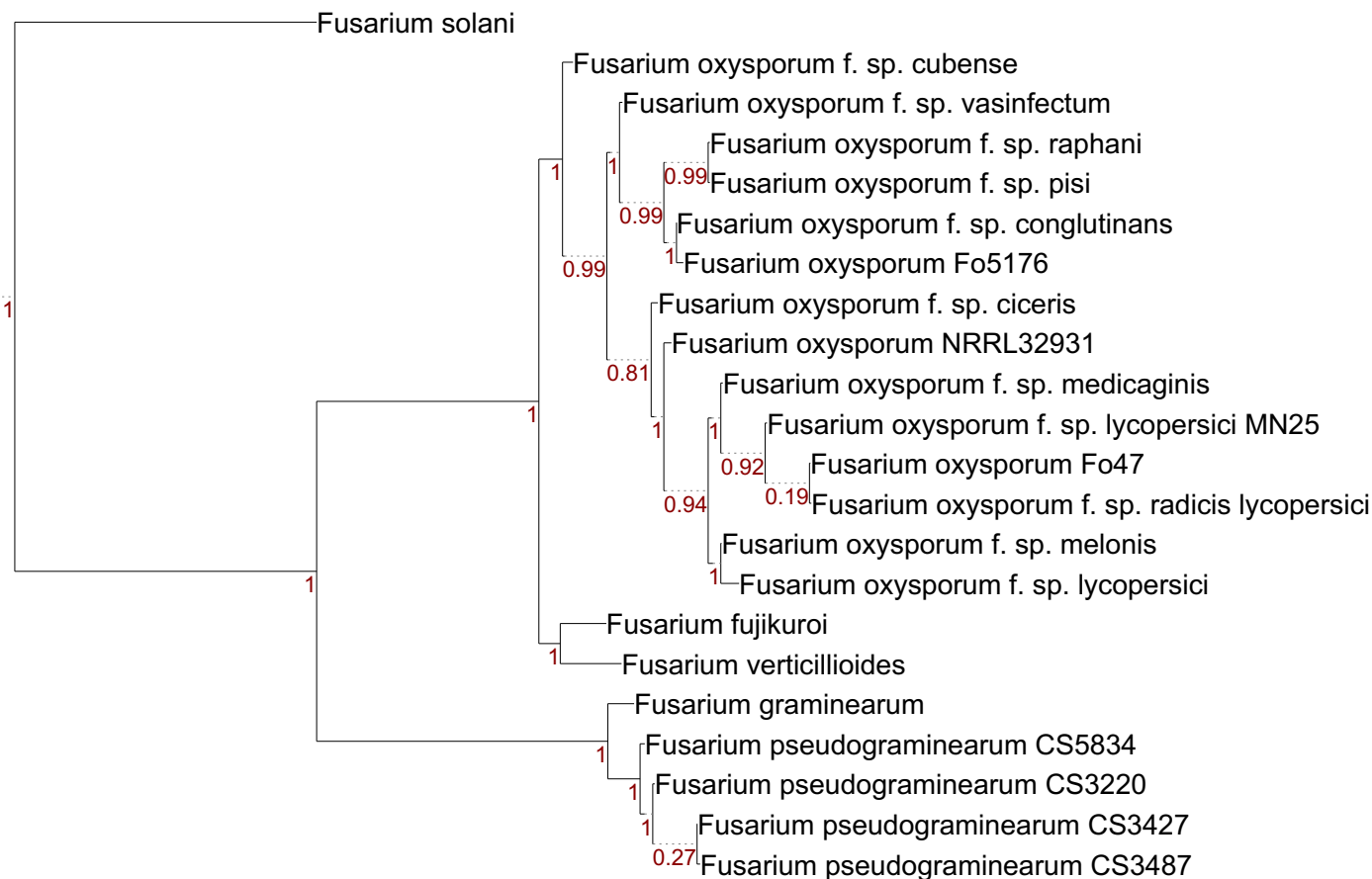

0.03

Supplement: Additional file 11: — Phylogenetic tree illustrating the relationship between the Fusarium sp. isolates used in this study Branch support values are shown in red. This tree illustrates that the legume-infecting isolates do not appear to be more closely related to each other than to other ff. spp. (PDF 21 kb) [file 12864_2016_2486_MOESM11_ESM.pdf]

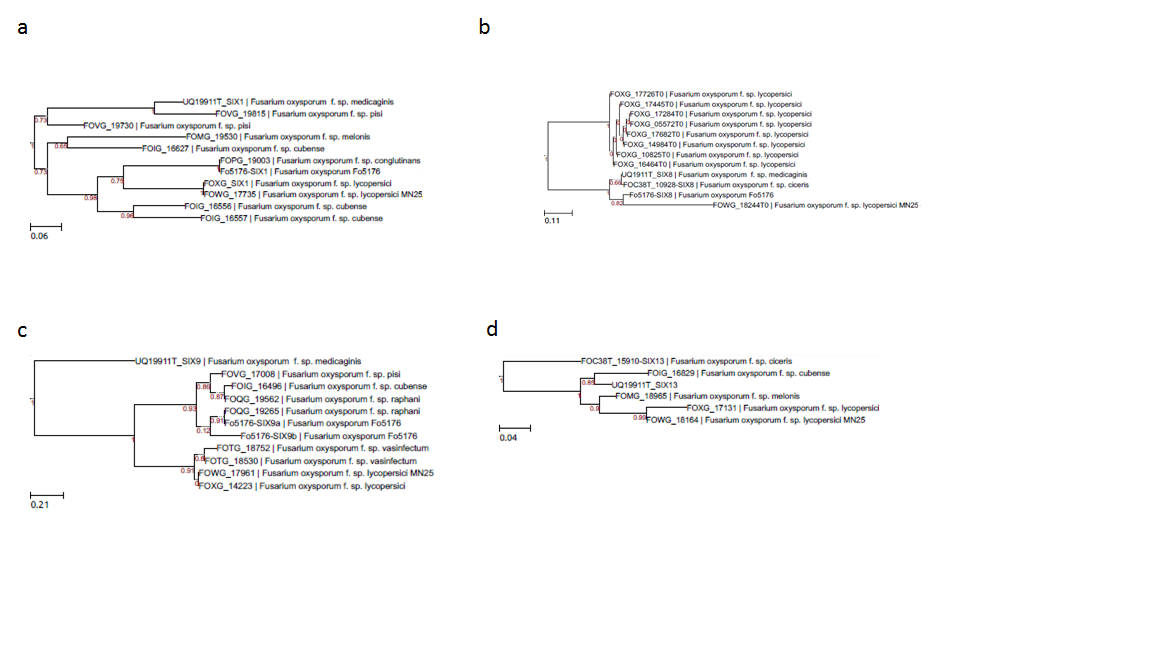

Supplement: Additional file 24: — Phylogenetic trees illustrating the relationship between SIX proteins detected as encoded in Fusarium oxysporum f. sp. medicaginis and other ff. spp. (a) SIX1, (b) SIX8, (c) SIX9 and (d) SIX13. The relationship between the SIX proteins suggests a greater similarity between those from the legume–infecting ff. spp. than the phylogenetic analysis based on core proteins identified. (PNG 165 kb) [file 12864_2016_2486_MOESM24_ESM.png]

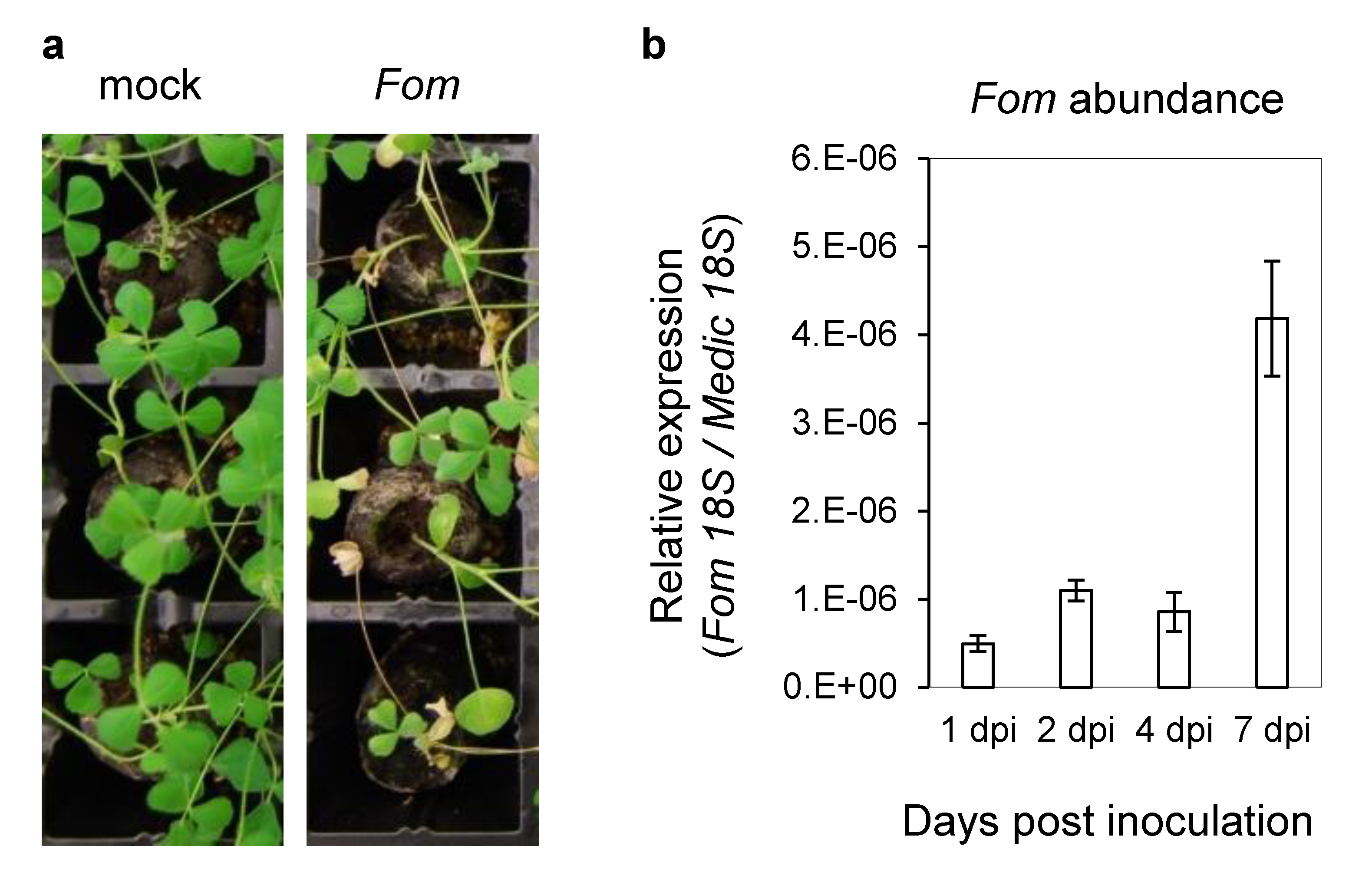

Supplement: Additional file 25: — Disease symptoms and relative abundance of Fom -5190a in infected M. truncatula DZA315 root samples. (a) Disease symptoms of DZA315 plants at 14 days post treatment with Fom-5190a or a control (mock) treatment. (b) Relative Fom-5190a fungal abundance was determined by qRT-PCR expression of Fom-5190a_18S relative to M. truncatula_18S expression in M. truncatula DZA315 root samples harvested at 1, 2, 4 and 7 days post inoculation (dpi). Samples are averages ± SE of 4 biological replicates consisting of pools of 10 seedlings. (TIF 2196 kb) [file 12864_2016_2486_MOESM25_ESM.tif]
