# Supplementary material for: Comparative genomics and prediction of conditionally dispensable sequences in legume–infecting Fusarium oxysporum formae speciales facilitates identification of candidate effectors
Source: BMC Genomics. 2016 Mar 5;17:191. doi: 10.1186/s12864-016-2486-8 (PMC4779268; doi:10.1186/s12864-016-2486-8)
Supplement: Additional file 3: — Sequencing data used for genome assemblies. (DOCX 13 kb) [file 12864_2016_2486_MOESM3_ESM.docx]

**Additional File 3 Sequencing data used for genome assemblies.**

| **Isolate** | **Trimmed data used for assembly (Gb)** | **Insert size** | **Sequencing type** | **Service Provider** |
| --- | --- | --- | --- | --- |
|  |  |  |  |  |
| *Fom*-5190a | 8.28 | 200 bp | Paired end Illumina | CSIRO/The University of Western Australia |
| *Fom*-5190a | 0.18 | 500 bp | Paired-end Illumina | CSIRO/The University of Western Australia |
| *Fom*-5190a | 0.01 | 3 kb | Mate-paired 454 | The Australian National University |
| *Fom*-5190a | 0.8 | 3 kb | Long jumping distance, Illumina | Eurofins, Luxemborg |
| *Fom*-5190a | 0.21 | 8 kb | Long jumping distance, Illumina | Eurofins, Luxemborg |
| *Foc*38-1 | 13.6 | 200 bp | Paired-end Illumina | Xcelris genomics, India |
| *Foc*38-1 | 9.7 | 2 kb | Mate-paired Illumina | Xcelris genomics, India |
| *Foc*38-1 | 8.3 | 5 kb | Mate-paired Illumina | Xcelris genomics, India |
| *Fop*-37622 | 7.9 | 180 bp | Mate-paired Illumina | Broad Institute, USA |
| *Fop-*37622 | 5.3 | 3 kb | Long jumping distance, Illumina | Broad Institute, USA |
