# Supplementary material for: Comparative genomics and prediction of conditionally dispensable sequences in legume–infecting Fusarium oxysporum formae speciales facilitates identification of candidate effectors
Source: BMC Genomics. 2016 Mar 5;17:191. doi: 10.1186/s12864-016-2486-8 (PMC4779268; doi:10.1186/s12864-016-2486-8)
Supplement: Additional file 4: — Protein/gene set comparisons across Fusarium sp. (DOCX 11 kb) [file 12864_2016_2486_MOESM4_ESM.docx]

**Additional File 4 Protein/gene set comparisons across *Fusarium* sp.**

|  | ***Foc*** | ***Fom*** | ***Fop*** |
| --- | --- | --- | --- |
| **Total number of predicted proteins** | 16,124 | 16,858 | 19,623 |
| **Average protein length (aa)** | 462 | 457 | 432 |
| **Maximum protein length (aa)** | 7,584 | 7,507 | 7,755 |
| **Minimum protein length (aa)^b^** | 35 | 32 | 30 |
| **tRNAs decoding standard 20 aa** | 288 | 297 | 318 |
| **Proteins with GO annotation** | 8,635 (54%) | 9,853 (58%) | 11,977 (61%) |
| **Proteins with assigned KEGG enzyme codes** | 2,590 | 2,908 | 2,703 |
| **Proteins with Pfam domains** | 11,121 (69%) | 11,468 (68%) | 12,303 (63%) |
