# Supplementary material for: Comparative genomics and prediction of conditionally dispensable sequences in legume–infecting Fusarium oxysporum formae speciales facilitates identification of candidate effectors
Source: BMC Genomics. 2016 Mar 5;17:191. doi: 10.1186/s12864-016-2486-8 (PMC4779268; doi:10.1186/s12864-016-2486-8)
Supplement: Additional file 12: — Ortholog found only in legume-infecting Fusarium sp. (DOCX 11 kb) [file 12864_2016_2486_MOESM12_ESM.docx]

The specificity of orthologs to certain f. sp. or species, or to *Fusarium* spp. collectively, were used as criteria in prediction of *Fom*-5190a pathogenicity genes as we hypothesized that legume-infecting *F. oxysporum* ff. spp. may share some common strategies in infection processes highlighted by the presence of similar pathogenicity proteins. However, we found only one ortholog group exclusively amongst the legume-infecting *Fusarium* spp. (*Fom*-5190a*, Foc*-38-1, *Fop-*37622 and *F. solani*). The orthologous proteins had close similarity to poly(3-hydroxybutyrate) depolymerases found in other Sordariomycetes species but not in other *Fusarium* species. The best BLASTP match for this protein in *Fom*-5190a (FOXM_5190a_16175*,* encoded on predicted dispensable scaffold 256), was an identical protein from *Fop*-37622, although the *Fom*-5190a ortholog was not detected as expressed *in planta* under the conditions examined (2dpi).
