# Supplementary material for: Comparative genomics and prediction of conditionally dispensable sequences in legume–infecting Fusarium oxysporum formae speciales facilitates identification of candidate effectors
Source: BMC Genomics. 2016 Mar 5;17:191. doi: 10.1186/s12864-016-2486-8 (PMC4779268; doi:10.1186/s12864-016-2486-8)
Supplement: Additional file 14: — Comparison of differences in length between core and lineage specific scaffolds across Fusarium species. (DOCX 12 kb) [file 12864_2016_2486_MOESM14_ESM.docx]

Additional File 14. Comparison of differences in length between core and lineage specific scaffolds across *Fusarium* species.

| **Isolate** **Chromosome** | **# Scaffold Sequences** | **Total Length** | **Avg. Length** |
| --- | --- | --- | --- |
| ***Fom*-5190a core** | 446 | 42,167,838 | 94,547 |
| **dispensable** | 3,529 | 8,766,042 | 2,484 |
| ***Foc*-38-1 core** | 413 | 41,518,444 | 100,529 |
| **dispensable** | 1,069 | 13,294,565 | 12,436 |
| ***Fop-*37622 core** | 116 | 43,587,736 | 375,756 |
| **dispensable** | 356 | 11,600,480 | 64,988 |
| ***Fol* core** | 11 | 43,414,532 | 3,946,776 |
| **dispensable** | 4 | 14,306,028 | 3,576,507 |
| ***F. solani* core** | 11 | 30,178,653 | 2,743,514 |
| **dispensable** | 3 | 1,837,557 | 612,519 |
