# Supplementary material for: Comparative genomics and prediction of conditionally dispensable sequences in legume–infecting Fusarium oxysporum formae speciales facilitates identification of candidate effectors
Source: BMC Genomics. 2016 Mar 5;17:191. doi: 10.1186/s12864-016-2486-8 (PMC4779268; doi:10.1186/s12864-016-2486-8)
Supplement: Additional file 15: — Pfam domains more abundant on predicted dispensable scaffolds in Fom -5190a. (DOCX 18 kb) [file 12864_2016_2486_MOESM15_ESM.docx]

**Additional File 15. Pfam domains more abundant on predicted dispensable scaffolds in *Fom*-5190a.**

| Pfam domain description | Pfam number | Total number of genes with domain | Number  of genes with domain on predicted dispensable scaffolds | P-value |
| --- | --- | --- | --- | --- |
| 3-beta hydroxysteroid dehydrogenase/isomerase family | PF01073 | 85 | 54 | 9.94E-03 |
| Zinc-binding dehydrogenase | PF00107 | 124 | 78 | 4.01E-03 |
| Flavin containing amine oxidoreductase | PF01593 | 46 | 30 | 2.73E-02 |
| AMP-binding enzyme | PF00501 | 78 | 49 | 1.38E-02 |
| Ankyrin repeat | PF00023 | 161 | 95 | 5.74E-03 |
| Carboxylesterase family | PF00135 | 78 | 58 | 1.13E-03 |
| Fungal specific transcription factor domain | PF04082 | 402 | 221 | 1.78E-03 |
| Metallo-beta-lactamase superfamily | PF00753 | 32 | 21 | 4.59E-02 |
| Pyridine nucleotide-disulphide oxidoreductase | PF00070 | 69 | 47 | 7.09E-03 |
| Amino acid permease | PF00324 | 97 | 58 | 1.54E-02 |
| alpha/beta hydrolase fold | PF00561 | 83 | 52 | 1.24E-02 |
| short chain dehydrogenase | PF00106 | 296 | 176 | 5.02E-04 |
| Chitin recognition protein | PF00187 | 12 | 11 | 3.98E-02 |
| Condensation domain | PF00668 | 21 | 16 | 3.85E-02 |
| FAD dependent oxidoreductase | PF01266 | 147 | 83 | 1.39E-02 |
| NAD dependent epimerase/dehydratase family | PF01370 | 162 | 97 | 4.31E-03 |
| Fumarylacetoacetate (FAA) hydrolase family | PF01557 | 18 | 14 | 4.46E-02 |
| FAD binding domain | PF00890 | 92 | 53 | 2.46E-02 |
| FAD binding domain | PF01494 | 108 | 72 | 2.25E-03 |
| Flavin-binding monooxygenase-like | PF00743 | 59 | 39 | 1.49E-02 |
| Glycosyl hydrolase family 3 N terminal domain | PF00933 | 38 | 26 | 2.68E-02 |
| Glycosyl hydrolase family 3 C-terminal domain | PF01915 | 36 | 25 | 2.65E-02 |
| Glycosyl hydrolases family 43 | PF04616 | 37 | 26 | 2.30E-02 |
| GMC oxidoreductase | PF05199 | 34 | 23 | 3.50E-02 |
| GMC oxidoreductase | PF00732 | 35 | 24 | 3.05E-02 |
| Methyltransferase small domain | PF05175 | 23 | 17 | 3.91E-02 |
| NADH:flavin oxidoreductase / NADH oxidase family | PF00724 | 27 | 19 | 3.99E-02 |
| Cytochrome P450 | PF00067 | 163 | 100 | 2.60E-03 |
| X-Pro dipeptidyl-peptidase (S15 family) | PF02129 | 22 | 16 | 4.59E-02 |
| Subtilase family | PF00082 | 41 | 25 | 4.96E-02 |
| Prolyl oligopeptidase family | PF00326 | 47 | 28 | 4.78E-02 |
| Phosphorylase superfamily | PF01048 | 28 | 20 | 3.43E-02 |
| Phosphopantetheine attachment site | PF00550 | 36 | 25 | 2.65E-02 |
| Sugar (and other) transporter | PF00083 | 331 | 193 | 5.70E-04 |
| ubiE/COQ5 methyltransferase family | PF01209 | 40 | 26 | 3.51E-02 |
| Fungal Zn(2)-Cys(6) binuclear cluster domain | PF00172 | 485 | 275 | 2.43E-04 |
| NmrA-like family | PF05368 | 129 | 82 | 2.90E-03 |
| NACHT domain | PF05729 | 90 | 56 | 1.12E-02 |
| Heterokaryon incompatibility protein (HET) | PF06985 | 149 | 85 | 1.17E-02 |
| Tannase and feruloyl esterase | PF07519 | 14 | 12 | 4.20E-02 |
| Major Facilitator Superfamily | PF07690 | 613 | 371 | 7.53E-07 |
| alpha/beta hydrolase fold | PF07859 | 113 | 78 | 9.12E-04 |
| Pyridine nucleotide-disulphide oxidoreductase | PF07992 | 118 | 67 | 1.91E-02 |
| Male sterility protein | PF07993 | 57 | 33 | 4.49E-02 |
| Alcohol dehydrogenase GroES-like domain | PF08240 | 116 | 73 | 4.89E-03 |
| Methyltransferase domain | PF08241 | 115 | 72 | 5.45E-03 |
| Methyltransferase domain | PF08242 | 68 | 44 | 1.35E-02 |
| KR domain | PF08659 | 225 | 137 | 8.73E-04 |
| Fungal specific transcription factor domain | PF11951 | 163 | 100 | 2.60E-03 |
| Alpha/beta hydrolase family | PF12695 | 153 | 88 | 9.86E-03 |
| Alpha/beta hydrolase family | PF12697 | 180 | 104 | 6.20E-03 |
| Ankyrin repeats (3 copies) | PF12796 | 163 | 96 | 5.73E-03 |
| Methyltransferase domain | PF12847 | 101 | 66 | 4.05E-03 |
| AMP-binding enzyme C-terminal domain | PF13193 | 50 | 31 | 3.44E-02 |
| L-lysine 6-monooxygenase (NADPH-requiring) | PF13434 | 72 | 44 | 2.14E-02 |
| NAD(P)-binding Rossmann-like domain | PF13450 | 165 | 100 | 3.21E-03 |
| NADH(P)-binding | PF13460 | 174 | 102 | 5.22E-03 |
| Methyltransferase domain | PF13489 | 122 | 79 | 2.48E-03 |
| Amino acid permease | PF13520 | 100 | 60 | 1.39E-02 |
| Enoyl-(Acyl carrier protein) reductase | PF13561 | 157 | 95 | 3.88E-03 |
| Zinc-binding dehydrogenase | PF13602 | 47 | 28 | 4.78E-02 |
| Ankyrin repeat | PF13606 | 156 | 93 | 5.22E-03 |
| Ankyrin repeats (many copies) | PF13637 | 153 | 93 | 3.86E-03 |
| Methyltransferase domain | PF13649 | 48 | 32 | 2.11E-02 |
| Pyridine nucleotide-disulphide oxidoreductase | PF13738 | 97 | 60 | 1.02E-02 |
| Methyltransferase domain | PF13847 | 98 | 65 | 3.50E-03 |
| Ankyrin repeats (many copies) | PF13857 | 141 | 85 | 5.70E-03 |
| Fibronectin type III-like domain | PF14310 | 31 | 21 | 4.02E-02 |
