# Supplementary material for: Comparative genomics and prediction of conditionally dispensable sequences in legume–infecting Fusarium oxysporum formae speciales facilitates identification of candidate effectors
Source: BMC Genomics. 2016 Mar 5;17:191. doi: 10.1186/s12864-016-2486-8 (PMC4779268; doi:10.1186/s12864-016-2486-8)
Supplement: Additional file 16: — Pfam domains more abundant on predicted dispensable scaffolds in Foc -38-1. (DOCX 14 kb) [file 12864_2016_2486_MOESM16_ESM.docx]

**Additional File 16. Pfam domains more abundant on predicted dispensable scaffolds in *Foc*-38-1.**

| **Pfam domain description** | **Pfam number** | **Total number of genes with domain** | **Number of genes with domain on predicted dispensable scaffolds** | **P-value** |
| --- | --- | --- | --- | --- |
| Ankyrin repeats (3 copies) | PF12796 | 189 | 74 | 4.66E-07 |
| Ankyrin repeat | PF00023 | 188 | 72 | 1.21E-06 |
| NACHT domain | PF05729 | 109 | 48 | 3.67E-06 |
| Ankyrin repeats (many copies) | PF13637 | 182 | 68 | 4.30E-06 |
| Ankyrin repeat | PF13606 | 183 | 68 | 4.94E-06 |
| Ankyrin repeats (many copies) | PF13857 | 164 | 62 | 7.99E-06 |
| Reverse transcriptase (RNA-dependent DNA polymerase) | PF07727 | 15 | 15 | 1.65E-05 |
| Integrase core domain | PF00665 | 13 | 13 | 5.96E-05 |
| DDE superfamily endonuclease | PF03184 | 23 | 17 | 6.05E-05 |
| Helitron helicase-like domain at N-terminus | PF14214 | 15 | 13 | 1.50E-04 |
| Protein of unknown function (DUF3505) | PF12013 | 11 | 11 | 2.17E-04 |
| MULE transposase domain | PF10551 | 20 | 14 | 3.75E-04 |
| PIF1-like helicase | PF05970 | 14 | 11 | 8.05E-04 |
| Tetratricopeptide repeat | PF13374 | 32 | 17 | 8.95E-04 |
| bZIP transcription factor | PF00170 | 40 | 19 | 1.23E-03 |
| Phosphorylase superfamily | PF01048 | 28 | 15 | 1.60E-03 |
| HNH endonuclease | PF13391 | 11 | 9 | 1.95E-03 |
| Reverse transcriptase (RNA-dependent DNA polymerase) | PF00078 | 17 | 11 | 2.28E-03 |
| NB-ARC domain | PF00931 | 21 | 12 | 3.01E-03 |
| Patatin-like phospholipase | PF01734 | 26 | 13 | 4.61E-03 |
| GAG-pre-integrase domain | PF13976 | 6 | 6 | 6.01E-03 |
| Glycosyl hydrolases family 18 | PF00704 | 28 | 13 | 7.01E-03 |
| Helix-loop-helix DNA-binding domain | PF00010 | 26 | 12 | 9.43E-03 |
| Protein of unknown function (DUF3295) | PF11702 | 5 | 5 | 1.20E-02 |
| Heterokaryon incompatibility protein (HET) | PF06985 | 146 | 40 | 1.30E-02 |
| hAT family C-terminal dimerisation region | PF05699 | 28 | 12 | 1.36E-02 |
| Chromo (CHRromatin Organisation MOdifier) domain | PF00385 | 21 | 10 | 1.41E-02 |
| Tc5 transposase DNA-binding domain | PF03221 | 21 | 10 | 1.41E-02 |
| Putative phosphatase regulatory subunit | PF03370 | 6 | 5 | 1.84E-02 |
| Protein of unknown function (DUF3723) | PF12520 | 6 | 5 | 1.84E-02 |
| Est1 DNA/RNA binding domain | PF10373 | 6 | 5 | 1.84E-02 |
| FAR1 DNA-binding domain | PF03101 | 6 | 5 | 1.84E-02 |
| Tetratricopeptide repeat | PF07721 | 6 | 5 | 1.84E-02 |
| Ulp1 protease family, C-terminal catalytic domain | PF02902 | 9 | 6 | 1.91E-02 |
| DDE superfamily endonuclease | PF13358 | 4 | 4 | 2.44E-02 |
| Retrotransposon gag protein | PF03732 | 4 | 4 | 2.44E-02 |
| Fungal protein of unknown function (DUF1752) | PF08550 | 7 | 5 | 2.64E-02 |
| Basic region leucine zipper | PF07716 | 21 | 9 | 2.80E-02 |
| Tetratricopeptide repeat | PF13424 | 54 | 17 | 2.85E-02 |
| BTB/POZ domain | PF00651 | 34 | 12 | 3.26E-02 |
| Domain of unknown function DUF221 | PF02714 | 11 | 6 | 3.30E-02 |
| Protein of unknown function (DUF3435) | PF11917 | 8 | 5 | 3.59E-02 |
| Endonuclease-reverse transcriptase | PF14529 | 8 | 5 | 3.59E-02 |
| Tetratricopeptide repeat | PF13431 | 32 | 11 | 4.22E-02 |
| LysM domain | PF01476 | 16 | 7 | 4.38E-02 |
| Lysophospholipase catalytic domain | PF01735 | 9 | 5 | 4.67E-02 |
| Zinc-finger double domain | PF13465 | 38 | 12 | 4.99E-02 |
