# Supplementary material for: Comparative genomics and prediction of conditionally dispensable sequences in legume–infecting Fusarium oxysporum formae speciales facilitates identification of candidate effectors
Source: BMC Genomics. 2016 Mar 5;17:191. doi: 10.1186/s12864-016-2486-8 (PMC4779268; doi:10.1186/s12864-016-2486-8)
Supplement: Additional file 17: — Pfam domains more abundant on predicted dispensable scaffolds in Fop -37622. (DOCX 16 kb) [file 12864_2016_2486_MOESM17_ESM.docx]

**Additional File 17. Pfam domains more abundant on predicted dispensable scaffolds in *Fop*-37622.**

| **Pfam domain description** | **Pfam number** | **Total number of genes with domain** | **Number of genes with domain on predicted dispensable scaffolds** | **P-value** |
| --- | --- | --- | --- | --- |
| Alcohol dehydrogenase GroES-like domain | PF08240 | 129 | 28 | 4.35E-02 |
| Amidase | PF01425 | 51 | 14 | 3.82E-02 |
| Ankyrin repeat | PF00023 | 150 | 36 | 1.49E-02 |
| Ankyrin repeats (3 copies) | PF12796 | 156 | 34 | 3.35E-02 |
| Ankyrin repeat | PF13606 | 109 | 25 | 3.77E-02 |
| Ankyrin repeats (many copies) | PF13637 | 126 | 28 | 3.85E-02 |
| Bacteriorhodopsin-like protein | PF01036 | 7 | 4 | 4.92E-02 |
| BAH domain | PF01426 | 10 | 6 | 1.57E-02 |
| Right handed beta helix region | PF13229 | 11 | 6 | 2.08E-02 |
| bZIP transcription factor | PF00170 | 46 | 21 | 2.42E-04 |
| Basic region leucine zipper | PF07716 | 23 | 9 | 2.20E-02 |
| Putative phosphatase regulatory subunit | PF03370 | 7 | 6 | 5.40E-03 |
| Chromo (CHRromatin Organisation MOdifier) domain | PF00385 | 20 | 9 | 1.26E-02 |
| Chromo shadow domain | PF01393 | 6 | 5 | 1.17E-02 |
| DDE superfamily endonuclease | PF03184 | 11 | 10 | 2.64E-04 |
| DDE superfamily endonuclease | PF13358 | 3 | 3 | 3.84E-02 |
| Transposase IS4 | PF13843 | 8 | 7 | 2.52E-03 |
| hAT family C-terminal dimerisation region | PF05699 | 17 | 17 | 9.23E-07 |
| Fungal protein of unknown function (DUF1752) | PF08550 | 13 | 6 | 3.33E-02 |
| Protein of unknown function (DUF2421) | PF10334 | 10 | 5 | 4.06E-02 |
| Protein of unknown function (DUF3295) | PF11702 | 7 | 5 | 1.72E-02 |
| Protein of unknown function (DUF3435) | PF11917 | 6 | 5 | 1.17E-02 |
| Protein of unknown function (DUF3505) | PF12013 | 39 | 36 | 4.65E-12 |
| Protein of unknown function (DUF3533) | PF12051 | 7 | 4 | 4.92E-02 |
| Protein of unknown function (DUF3723) | PF12520 | 12 | 11 | 1.26E-04 |
| FAD binding domain | PF00890 | 59 | 19 | 7.74E-03 |
| FAD binding domain | PF01494 | 109 | 24 | 4.84E-02 |
| Putative FMN-binding domain | PF04299 | 8 | 5 | 2.38E-02 |
| Flavin-binding monooxygenase-like | PF00743 | 73 | 21 | 1.29E-02 |
| Frequency clock protein | PF09421 | 11 | 9 | 8.65E-04 |
| Fungal specific transcription factor domain | PF04082 | 455 | 82 | 4.32E-02 |
| Glycosyltransferase family 28 N-terminal domain | PF03033 | 13 | 7 | 1.38E-02 |
| Heterokaryon incompatibility protein (HET) | PF06985 | 160 | 36 | 2.49E-02 |
| Hydroxymethylglutaryl-coenzyme A reductase | PF00368 | 7 | 6 | 5.40E-03 |
| HNH endonuclease | PF13391 | 8 | 7 | 2.52E-03 |
| Hsp20/alpha crystallin family | PF00011 | 7 | 4 | 4.92E-02 |
| Tc5 transposase DNA-binding domain | PF03221 | 8 | 5 | 2.38E-02 |
| Methyltransferase domain | PF13489 | 131 | 31 | 2.22E-02 |
| Mis12-Mtw1 protein family | PF08202 | 6 | 5 | 1.17E-02 |
| NACHT domain | PF05729 | 109 | 34 | 1.09E-03 |
| NAD(P)-binding Rossmann-like domain | PF13450 | 157 | 34 | 3.49E-02 |
| NmrA-like family | PF05368 | 126 | 27 | 4.82E-02 |
| Periplasmic copper-binding protein (NosD) | PF05048 | 3 | 3 | 3.84E-02 |
| Cytochrome P450 | PF00067 | 184 | 47 | 3.72E-03 |
| Patatin-like phospholipase | PF01734 | 21 | 9 | 1.53E-02 |
| Calpain family cysteine protease | PF00648 | 12 | 7 | 1.05E-02 |
| Ulp1 protease family, C-terminal catalytic domain | PF02902 | 8 | 5 | 2.38E-02 |
| PIF1-like helicase | PF05970 | 8 | 7 | 2.52E-03 |
| Phosphorylase superfamily | PF01048 | 41 | 20 | 1.75E-04 |
| Pyridine nucleotide-disulphide oxidoreductase | PF07992 | 88 | 24 | 1.35E-02 |
| Pyridine nucleotide-disulphide oxidoreductase | PF13738 | 84 | 26 | 3.69E-03 |
| Regulator of G protein signaling domain | PF00615 | 11 | 6 | 2.08E-02 |
| Spherulation-specific family 4 | PF12138 | 4 | 4 | 1.67E-02 |
| Tetratricopeptide repeat | PF13374 | 27 | 11 | 1.06E-02 |
| Tetratricopeptide repeat | PF13424 | 21 | 8 | 3.17E-02 |
| Tetratricopeptide repeat | PF07721 | 5 | 4 | 2.57E-02 |
| Transketolase, thiamine diphosphate binding domain | PF00456 | 10 | 5 | 4.06E-02 |
| Tubulin/FtsZ family, GTPase domain | PF00091 | 10 | 5 | 4.06E-02 |
| Up-regulated During Septation | PF15456 | 9 | 7 | 3.82E-03 |
| Uncharacterised protein family (UPF0075) | PF03702 | 3 | 3 | 3.84E-02 |
| Zinc finger, C2H2 type | PF00096 | 82 | 21 | 2.69E-02 |
