# Supplementary material for: Comparative genomics and prediction of conditionally dispensable sequences in legume–infecting Fusarium oxysporum formae speciales facilitates identification of candidate effectors
Source: BMC Genomics. 2016 Mar 5;17:191. doi: 10.1186/s12864-016-2486-8 (PMC4779268; doi:10.1186/s12864-016-2486-8)
Supplement: Additional file 18: — Proteins with similarities to known fungal pathogenicity genes and their expression at 2 dpi in Fom -5190a. (DOCX 16 kb) [file 12864_2016_2486_MOESM18_ESM.docx]

Additional file 18. Proteins with similarities to known fungal pathogenicity genes and their expression at 2 dpi in *Fom*-5190a.

| **Protein class** | **Protein ortholog group** | **Scaffold (core / dispensable)** | ***Fom*-5190a expression i*n planta* at 2 dpi** | **Other details** |
| --- | --- | --- | --- | --- |
| **NLPs**  **(NPP1 domain)** | *FOXM*-*5190a_13982* | d | n | ortholog of 3 NLPs on Fol CDCs;FOXG_14409.2, FOXG_15072.2,FOXG_17014.2 |
|  | *FOXM*-*5190a_05475* | c | y | closest homology to NEP2 (PHI: 2712) a gene associated with reduced virulence in *V. dhaliae* |
|  | *FOXM*-*5190a_10883*  *FOXM*-*5190a_12494* | c  c | n  n |  |
|  | *FOXM*-*5190a_14391* | c | n |  |
|  | *FOC38_05220*  *FOC38_07236*  *FOC38_11834*  *FOC38_13786* | c  c  c  c | -  -  -  - |  |
| **Maackiain detoxifying** | *FOXM*-*5190aT_04021* | c | n | MAK1 like |
|  | *FOC38_15878* | d | - | MAK1 like, partial match |
| **Pisatin demethylase (PDA)** | *FOXM*-*5190a_16236*  *FOXM*-*5190a_16029*  *FOXM*-*5190a_13564* | c  d  d | y  y  y | PDA1 ortholog |
|  | *FOXM*-*5190a_12260* | c | n | PDA1 ortholog |
|  | *FOC38_12791*  *FOC38_09206* | d  d | -  - | PDA1 ortholog |
| **PEP2**  **Pea pathogenicity (PEP)** | *FOC38_09209* | d | - | PEP2 ortholog |
|  | *FOXM*-*5190a_13563*  *FOXM*-*5190a_15270* | d  d | y  y | PEP5 ortholog |
|  | *FOC38_09210* | d | - | PEP5 ortholog |
| **Contain cerato-platanin domain** | *FOXM*-*5190a_04705 FOXM*-*5190a_10461* | c  c | y  y | Cerato-platanin proteins found in the cell walls of fungi and are known to cause necrosis and induce phytoalexin production [[87](#_ENREF_87)]. They can also trigger plasmolysis, production of ROS and up-regulation of defence response genes in host and non-host plants (summarised in [[86](#_ENREF_86)]). |
|  | *FOXM*-*5190a_08816 FOXM*-*5190a_16529*  *FOXM*-*5190a_11981* | c  d  c | y  n  y |  |
|  | *FOXM*-*5190a_10339* | c | n |  |
|  | *FOXM*-*5190a_06293* | c | n |  |
|  | *FOC38_03383*  *FOC38_08708* | c  c | -  - |  |
|  | *FOC38_05538*  *FOC38_10247*  *FOC38_09039*  *FOC38_11628* | c  c  d  d | -  -  -  - |  |
|  | *FOC38_09459* | c | - |  |
