# Supplementary material for: Comparative genomics and prediction of conditionally dispensable sequences in legume–infecting Fusarium oxysporum formae speciales facilitates identification of candidate effectors
Source: BMC Genomics. 2016 Mar 5;17:191. doi: 10.1186/s12864-016-2486-8 (PMC4779268; doi:10.1186/s12864-016-2486-8)
Supplement: Additional file 22: — Best BLASTP matches of Fom -5190a candidate effector proteins and SIX protein orthologs versus NCBI non-redundant protein database (Feb 2015). (DOCX 15 kb) [file 12864_2016_2486_MOESM22_ESM.docx]

**Additional File 22** **Best BLASTP matches of *Fom*-5190a candidate effector proteins and SIX protein orthologs versus NCBI non-redundant protein database (Feb 2015).**

|  |  | **Best BLASTP match** | | | | | |  |
| --- | --- | --- | --- | --- | --- | --- | --- | --- |
| ***Fom* Protein ID** | **Length**  **(aa)** | **ID** | **Description** | **Species** | **Alignment** | **Identity (aa)** | **Notes** | **Best match in *Foc*-38-1** |
| FOXM-5190a_SIX1 | 284 | FOVG_19815 (EXA28588.1) | hypothetical protein | *Fusarium oxysporum* f. sp. *pisi* 37622 (HDV247) | 244/280 aa | 87% | Best matches are other *F. oxysporum* f. sp., top two in *Fop*-37622 | - |
| FOXM-5190a_SIX8 | 141 | secreted in xylem Six 8 (ACN69118.1) | secreted in xylem Six 8 | *Fusarium oxysporum* f. sp. *lycopersici* | 134/141 aa | 95% | Best matches are other *F. oxysporum* f. sp. | FOC38_10928-SIX8  140/141 aa 99% |
| FOXM-5190a_SIX9 | 123 | Six9 (AGG54050.1) | Six9 | *Fusarium oxysporum* f. sp. *lycopersici* Fol007 | 45/108 aa | 42% | Best matches are other *F. oxysporum* f. sp. | - |
| FOXM-5190a_SIX13 | 293 | FOMG_18965 (EXK24301.1) | hypothetical protein | *Fusarium oxysporum* f. sp. *melonis* 26406 | 229/307 aa | 74% | Next best match is *Fol* Six13 | FOC38_15910-SIX13-like  188/257 aa 73% |
| FOXM-5190a_15788 | 199 | FFUJ_03969 | uncharacterized protein | *F. fujikuroi* IMI 58289 | 89/189 aa | 45% | Top two hits are *F. fujikuroi* and *N. haematococca*, followed by other *F. oxysporum* f. sp.and Ascomycota | FOC38_15881  60/166 aa 36 % |
| FOXM-5190a_16235 | 265 | FOPG_19255 | hypothetical protein | *F. oxysporum* f. sp. *conglutinans* race 2 54008 | 230/263 aa | 87% | Best matches are other *F. oxysporum* f. sp. | - |
| FOXM-5190a_16257 | 91 | Cob_00676 | hypothetical protein | *Colletotrichum orbiculare* MAFF 240422 | 23/90 aa | 24% | Other best matches are plant pathogenic Ascomycota | - |
| FOXM-5190a_16301 | 144 | FOVG_19456 | hypothetical protein | *F. oxysporum* f. sp. *pisi-*37622 (HDV247) | 136/143 aa | 95% | Top 3 hits are *F. oxysporum* f. sp. followed by a range of bacteria and fungi sharing a common LysM motif. Includes the fungi *Bipolaris sorokiniana*, *Colletotrichum orbiculare*, *Leptosphaeria maculans*. | - |
| FOXM-5190a_16306 | 131 | FOC1_g10000425 | hypothetical protein | *F. oxysporum* f. sp. *cubense* race 1 | 116/128 aa | 91% | Next best matches are  *Colletotrichum* species | FOC38_16051  129/130aa 99% |
| FOXM-5190a_16326 | 111 | FOWG_17837 | hypothetical protein | *F. oxysporum* f. sp. *lycopersici* MN25 | 95/110 aa | 86% | Best matches are other *F. oxysporum* f. sp. | - |
