# Supplementary material for: Comparative genomics and prediction of conditionally dispensable sequences in legume–infecting Fusarium oxysporum formae speciales facilitates identification of candidate effectors
Source: BMC Genomics. 2016 Mar 5;17:191. doi: 10.1186/s12864-016-2486-8 (PMC4779268; doi:10.1186/s12864-016-2486-8)
Supplement: Additional file 23: — Best BLASTP matches of Foc 38-1 and Fop -37622 SIX protein orthologs and predicted Fop _SIX13 and [ _SIX14 aa sequences. (DOCX 18 kb) [file 12864_2016_2486_MOESM23_ESM.docx]

Additional File 23 Best BLASTP matches of *SIX* proteins from *Foc-*38-1 and *Fop-*37622 versus NCBI non-redundant protein database (Feb 2015) and other legume-infecting ff. spp.

| **Protein ID** | **Top Match ID** | **Description** | **Species** | **Alignment** | **Identity (aa)** | **Notes** | **Top match in *Fom*-5190a** | **Top match in*Fop*-37622** |
| --- | --- | --- | --- | --- | --- | --- | --- | --- |
| FOC38_15967-SIX5-like | FFUJ_14914 (CCT67942.1) | uncharacterized protein | *F. fujikuroi* IMI 58289 | 112/133 aa | 74% | Top two hits are *F. fujikuroi* and Fo5176 | - | - |
| FOC38_10928-SIX8 | Fol SIX 8 (ACN69118.1) | secreted in xylem Six8 | *Fusarium oxysporum* f. sp. *lycopersici* | 133/141 aa | 94% | Best matches are other *F. oxysporum* f. sp. | FOXM5190aT_SIX8  141/142 99% | - |
| FOC38_SIX11 | Six11 (AGG54052.1) | Six11 | *Fusarium oxysporum* f. sp. *lycopersici* Fol007 | 106/111aa | 98% | Next best matches are *Colletotrichum* sp. | - | - |
| FOC38_15910-SIX13-like | *FOM*G_18965 (EXK24301.1) | hypothetical protein | *Fusarium oxysporum* f. sp. *melonis* 26406 | 229/249 aa | 92% | Next best match is *Fol* Six13 | FOXM5190aT_SIX13 188/257 73% | FOP_SIX13  234/239 94% |
| FOC38_SIX14 | Six14 (AGG54055.1) | Six14 | *Fusarium oxysporum* f. sp. *lycopersici* Fol007 | 81/90 aa | 90% | Next best match is an *F. verticillioides* protein followed by several *Colletotrichum* sp. | - | FOP_SIX14  84/91 92% |
| FOVG_19815T0_SIX1 | FOVG_19815T0  (EXA31686.1) | self |  |  |  | Next best match *F.oxysporum* f. sp. *cubense* | FOXM5190aT_SIX1 245/281 87% | - |
| FOVG_19730T0_SIX1 | FOVG_19730T0 (EXA28676.1) | self |  |  |  | Next best match *F.oxysporum* f. sp. *cubense* | FOXM5190aT_SIX1 219/284 77% | - |
| FOVG_17008T0_SIX9 | FOVG_17008T0  (EXA28588.1) | self |  |  |  | Protein N-terminus matches to Fo5176 SIX9a | FOXM5190aT_SIX9 46/116 40% | FOC38T_13972  51/80 64% |
| FOP_SIX13 | FOMG_18965 (EXK24301.1) | hypothetical protein | *Fusarium oxysporum* f. sp. *melonis* 26406 | 260/293 | 89% | Next best match is *Fol* SIX13, a Verticillium protein and other ff. spp. | FOXM5190aT_SIX13 228/300 76% | FOC38T_15910-SIX13-like 234/249 94% |
| FOP_SIX14 | gb\|AGG54055.1\| | Six14 | *Fusarium oxysporum* f. sp. *lycopersici* Fol007 | 75/89 | 85% | Next best match to *F. verticilliodes* | - | FOC38T_SIX14  84/91 92% |

>FOP_SIX13

MTRFHLILLPLLFSWFSYCLGELEVSDLSDQPPSVENTYRDQAFNEEELLKVVDELSVKLTNHTERTLVSEAALQRRQNGQYPNGVCPRGGRFYFDIDEDTSCNAKWGIATAHETRTFGSTGSVCAGPFRRITCACCFTTYPITDNDRMDGIYCPKWEVCKQEPQRWSKWGKLVPHTSCVQAKKLTEILIATKKVVKEYCTPKRWLPSTGKGKNAKFHAWAYNYSTGQLTKLKWMYLKLDGQYAKSASGISEWGLTYSVNEHNAIELCGYPSDDMQKNSIDAELQWEVTLQ*

>FOP_SIX14

MHFEYLLLLLVPTGALSQRILGCRMPNGSLNPSPNICNQAGGSFRSESVRVCCTRNNRDGPGVTESRFISGCNDNGGFVGSNEILATSC*
