# Supplementary material for: Comparative genomics and prediction of conditionally dispensable sequences in legume–infecting Fusarium oxysporum formae speciales facilitates identification of candidate effectors
Source: BMC Genomics. 2016 Mar 5;17:191. doi: 10.1186/s12864-016-2486-8 (PMC4779268; doi:10.1186/s12864-016-2486-8)
Supplement: Additional file 26: — Primer sequences used for qRT-PCR. (DOCX 12 kb) [file 12864_2016_2486_MOESM26_ESM.docx]

Additional File 26 **Primer sequences used for qRT-PCR.**

| **Primer name** | **Primer Sequence** |
| --- | --- |
| *Fom*-5190a_SIX1-F | AATCGCCTGGGACCTTTAAT |
| *Fom*-5190a_SIX1-R | GCTGAACCCCTCTCCATTC |
| *Fom*-5190a_SIX8-F | CGTTTCAGGCATATCAAGGA |
| *Fom*-5190a_SIX8-R | CTCCGCCCCTAGTTCTGTC |
| *Fom*-5190a_SIX9-F | CTGCTGCACCGATAATGAGA |
| *Fom*-5190a_SIX9-R | TGAACCCTCGAACGCTTACT |
| *Fom*-5190a_SIX13-F | ATTACAGCACGGGACAGCTT |
| *Fom*-5190a_SIX13-R | TGTAGGTGAGTCCCCATTCC |
| *Fom*-5190a_07571-F | GTTTCCTCCCGAATTGATGA |
| *Fom*-5190a_07571-R | CTGTGCTGATGCGAGAAGAG |
| *Fom*-5190a_15294-F | GCTGTACGCCTGGAACCTAC |
| *Fom*-5190a_15294-R | GTCTTCGGGGGACAGACA |
| *Fom*-5190a_15788-F | CGAAGCAGCAGGTTTATGTG |
| *Fom*-5190a_15788-R | ATTTCCCATCCCTTCGTTTT |
| *Fom*-5190a_16257-F | AGAAGGCGGAGCACACAC |
| *Fom*-5190a_16257-R | CGCAATGCCCGTATTACA |
| *Fom*-5190a_16301-F | TAGGCCTGGCATGAGTGTTA |
| *Fom*-5190a_16301-R | CCTAGAGAGTCGCCCTTCAT |
| *Fom*-5190a_16306-F | GACGATGGCGAGTACGTATG |
| *Fom*-5190a_16306-R | TTTAATACGGCCCAGATCCTT |
| *Fom*-5190a_16325-F | GCTAGTGTGTCTGGCCATGT |
| *Fom*-5190a_16325-R | TCAGTTGGAAGCCGTGTAGA |
| *Fom*-5190a_16326-F | CTGTGGCAGTGGTAAACACC |
| *Fom*-5190a_16326-R | GATAAAATTCGGGGCCTTG |
| *Fom*-5190a_13365-Actin-F | CACCACCTTTAACTCCATCA |
| *Fom-*5190a_13365-Actin-R | TCGGAGAGACCAGGGTACAT |
| *Fo_18s-F* | CGCCAGAGGACCCCTAAAC |
| *Fo_18s-R* | ATCGATGCCAGAACCAAGAGA |
| *M. truncatula*_B-tub-F | CCTGTTGCCGGTTCATAATC |
| *M. truncatula*_B-tub-F | CCCAAACATAGATTGCTGCTT |
| *M. truncatula_18s-F* | GCGCAAATTACCCAATCCTA |
| *M. truncatula_18s-R* | CCAACCCAAGGTCCAACTAC |
